# Catheters linked thrombosis in neonates: a single center observational study

**DOI:** 10.1186/s13052-024-01708-8

**Published:** 2024-08-13

**Authors:** Marwa Mohamed Farag, Hesham Abd El Rahim Ghazal, Mohamed Masoud Radwan, Nada Saeid El-sayed

**Affiliations:** 1 The division of neonatology, Pediatric department, Faculty of medicine, Alexandria University, Alexandria, Egypt; 2 Radiodoagnosis and intervention department, Faculty of medicine, Alexandria University, Alexandria, Egypt

**Keywords:** Central catheter, Thrombosis, PRBCs transfusion, Doppler scan

## Abstract

**Background:**

Central venous catheters (CVCs) are the major risk factors for neonatal thrombosis that might negatively affect morbidity and mortality in neonates. The aim of the present work was to estimate the incidence of CVC-linked thrombosis, among neonates in the NICU of Alexandria University Maternity Hospital, Egypt, over 1year, and to determine its possible risk factors.

**Methods:**

This observational cohort study involved 134 newborn infants born from July 2020 to July 2021with CVCs insertion during their hospital stay. Patients who had congenital anomalies, had thrombosis unrelated to the implantation of CVCs or died before 7 days of catheter placement were excluded from the analysis. The 134 neonates who met the study’s eligibility requirements had 142 CVCs inserted. Serial ultrasound and Doppler scans on site of venous insertion of catheters were performed.

**Results:**

Seventeen patients with catheter’s thrombosis (12%) were found during the placement of 142 catheters or 1615 CVCs’ days, resulting in an overall rate of 10.5 thrombotic events per 1000 catheters’ days. We constructed a logistic regression model to identify risk factors behind CVC-linked thrombosis. In univariate analysis, femoral central venous lines (CVLs), catheter dwell-time, sepsis, packed red cells (PRBCs) transfusions and low platelet count were risk factors for CVC-linked thrombosis. Nevertheless, only PRBCs transfusion was significant in the multivariate analysis, with OR and 95% confidence level 5.768 (1.013–32.836).

**Conclusion:**

Many factors should be considered in prediction of patients at risk of thrombosis including sepsis, femoral line insertion, low platelet count and PRBCs-transfusions. In our analysis, PRBCs-transfusion through peripheral intravenous lines (PIVs) was the strongest factor associated with CVC-linked thrombosis.

**Supplementary Information:**

The online version contains supplementary material available at 10.1186/s13052-024-01708-8.

## Background

Insertion of an intravascular catheter is the most common invasive procedure in the NICU and it is considered as a “life line” in neonatal care. The use of central venous catheters (CVCs) is not without risks, as they may result in mechanical injuries, infection, or thrombosis. CVCs are the most common cause of neonatal thrombotic events. Neonates are at greater risk for thrombosis owing to their immature hemostatic and coagulation systems, small blood vessel diameter, need for infusion of high-osmolar solutions, and low flow rate of infusate [1].

Abdominothoracic X-ray is the most frequently used method to define the position of the CVC tip and is regarded as the gold standard. Ultrasonography is increasingly used in realtime-CVCs insertion, control of the tip position with significantly less time and without exposure to ionizing radiation, and in early detection of CVC-linked thrombotic events. [2]

The aim of the present work was to estimate the incidence of CVC-linked thrombosis among neonates in the NICU of Alexandria University Maternity Hospital, Egypt, and to determine its possible risk factors.

## Methods

**Fig. 1:**
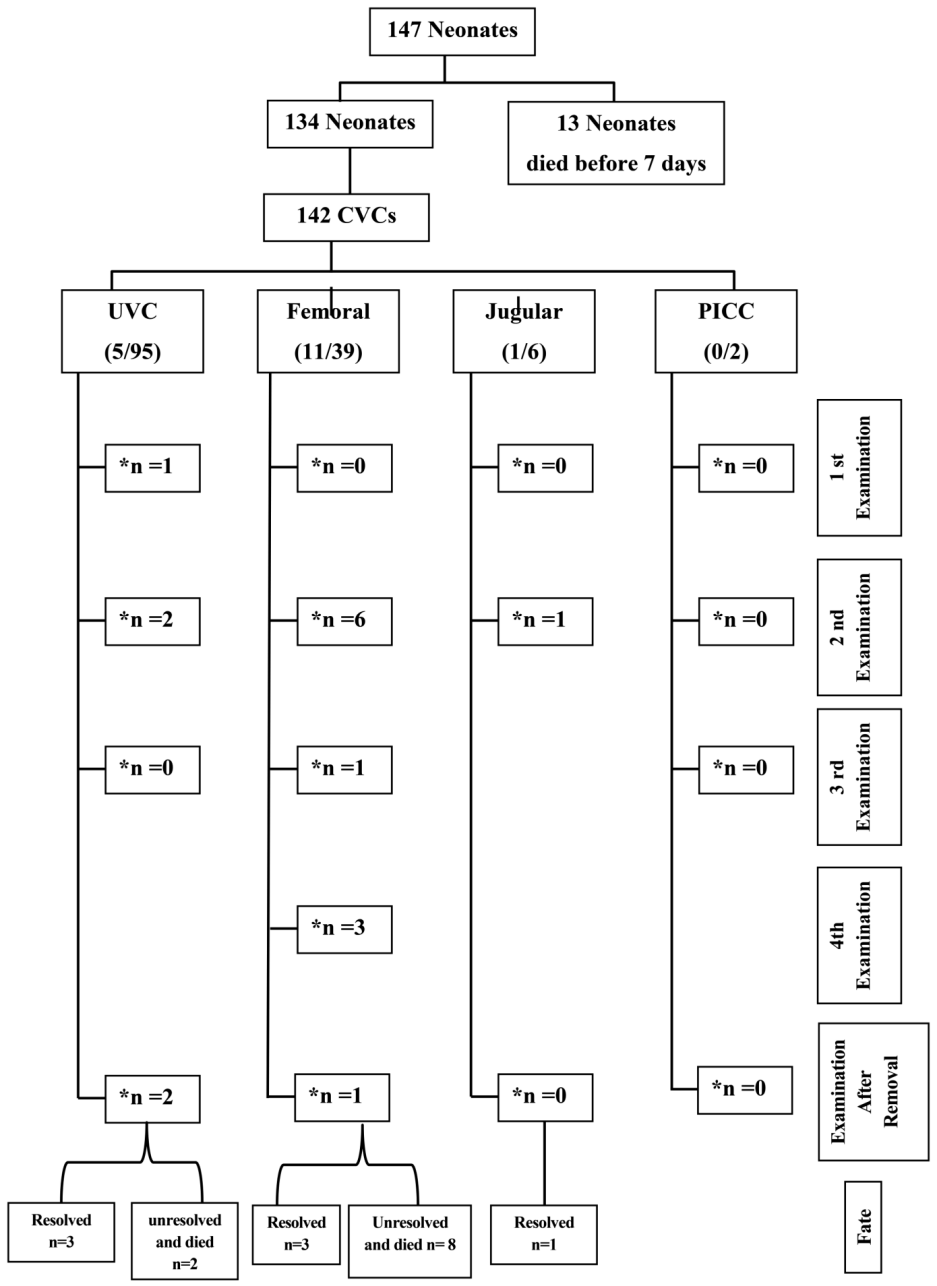
Flow chart of patients. *n= number of patients with de-novo thrombi at each examination

The work was of a prospective cohort design. Eligible neonates admitted to the NICU who had CVCs insertion whether umbilical venous catheter (UVC), peripherally inserted central catheter (PICC), or non-tunneled central venous line (NT-CVL) in the internal jugular vein or femoral vein were included in this research. The newly born patients with major congenital malformations or patients already admitted with thrombotic events not related to CVCs insertion were excluded from the analysis. Also, infants who died before 7 days of age were excluded from the analysis, Fig. 1.

Patients enrolled in this work (*n* = 134) were randomly assigned to the research protocol using computer-based system from total NICU’s admissions in the period under investigation (*n* = 1850 patients). The population was sampled by the simple random method until the required sample size was reached. The minimal sample size of 134 neonates to assess incidence of CVC-linked thrombotic episodes among neonates in the NICU of Alexandria University Maternity Hospital was calculated using PASS program version 20, based on a study assuming that CVC-linked thrombosis incidence rate is 10%, using a 95% confidence interval and 5% precision after adding 10% drop out rate.

UVC was the initial vascular access of choice in critically ill full term and preterm neonates. However, if umbilical venous cannulation failed or was removed for completing 14 days, [3] the recommended maximum time allowed for UVC dwelling, and patient was still in need for central line, then either a PICC or a NT-CVL, in femoral vein or internal jugular vein was inserted. Also, when it seemed likely that full enteral nutrition would not be possible for > 7 days (especially in premature infants), locally irritant drugs (e.g., inotropes) were needed, peripheral venous network had been exhausted, or exchange transfusion was demanded, then CVC was inserted. PICC was chosen in more stable infants who were not at need of frequent sampling.

Inserted UVCs (4–6 Fr.), PICCs (1 Fr.), and NT-CVCs (3 Fr.) were all of the Vygon type (Vygon, Écouen, France). We used single-lumen radiopaque catheters made of polyurethane.

The predetermined catheter length in case of UVC was determined using Shukla equation, catheter length = ([(wt×3) + 9]/2). The calculated catheter length was added up to the umbilical cord stump length to obtain the final insertion length.

NT-CVL in femoral veins or internal jugular veins were inserted using an aseptic technique, a puncture was made with real-time ultrasound guidance in short or long axis view or sometimes with landmark technique. After puncturing the vein and aspiration of venous blood to confirm venous puncture, a guidewire was inserted to guide the catheter placement. The catheter was introduced using Seldinger technique and was fixed with sutures.

Following CVCs insertion, the catheter’s tip position was verified using plain anteroposterior chest/abdomen x-ray and ultrasound. Once the CVCs were inserted, unfractionated heparin was continuously infused at a concentration of 0.5 unit/mL to maintain patency of CVCs. All blood products were administered through peripheral intravenous lines (PIVs).

Central line-associated blood stream infection (CLABSI) was defined by a laboratory confirmed bloodstream infection (BSI) in a neonate with a central catheter in situ for more than 48 h or within 24 h after CVC removal. That BSI was not related to an infection at any other site [4]. 

All neonates had serial radiological investigations either Doppler on the veins where the CVCs were inserted or portal vein Doppler for UVCs using GE vivid iq machine with 12 S-RS probe, frequency 5–11 Hz and GE 8 C-RS probe with a frequency range of 3.5–10 Hz for 2 D echocardiography.

Doppler ultrasound examination was performed within 24–48 h of catheter insertion and 7–10 days after catheter insertion. For surviving neonates, Doppler ultrasound examination was performed weekly until catheter withdrawal and within 48–72 h after catheter withdrawal. Another scan was done few days before discharge. If thrombosis was detected, ultrasound examination was done weekly until thrombosis resolution. The scans were performed by a single operator, and all images were revised by two consultants, a radiologist and a neonatologist experienced in targeted neonatal echocardiography, Faculty of Medicine.

For UVC, ultrasound examinations were performed via a thoraco-abdominal anterior approach (subcostal, abdominal, apical four-chamber, and sometimes parasternal short-axis views). The umbilical vein, left branch of portal vein, ductus venosus, umbilico-portal confluence, inferior vena cava (IVC), right atrium (RA), left atrium (LA) and right ventricle (RV) were recognized. The catheter appeared as an echogenic double-lined structure on longitudinal view and as a marked echogenic dot on transverse view. The position of the catheter tip was defined as the point at which the echogenic structure sharply disappeared. The adequate position was defined as the position of the catheter tip in the IVC or IVC/RA junction [5]. 

For NT-CVL ultrasound examination was done at site of the vein where the catheter was inserted extending to SVC or IVC, with visualization of cardiac chambers for intracardiac extension. A thrombus was defined as an echo dense structure within the heart or vessels around the catheter observed in two dimensions.

This work was performed in accordance with the ethical standards of the institutional research committee and with the 1964 Helsinki Declaration and its later amendments. The research protocol has been approved on 15th October 2020 by the Research Ethics Committee of Alexandria faculty of medicine. The approval no. was 0106559. Written informed consents were obtained from parents or authorized legal representatives of all newborns who participated in the study for having ultrasound and Doppler scans at different times and publication of anonymous patients’ data.

Data were fed to the computer and analyzed using IBM SPSS software package version 20.0. Qualitative data were described using number and percentage. Quantitative data were described using range (minimum and maximum), mean, standard deviation, median and interquartile range (IQR). Significance of the obtained results was judged at the 5% level. A univariate binary logistic regression analysis was done to test the effect of different variables on a given outcome. In the end, clinically and statistically significant variables were included in a multivariate analysis to find the most independent parameters related to this outcome.

## Results

**Fig. 2:**
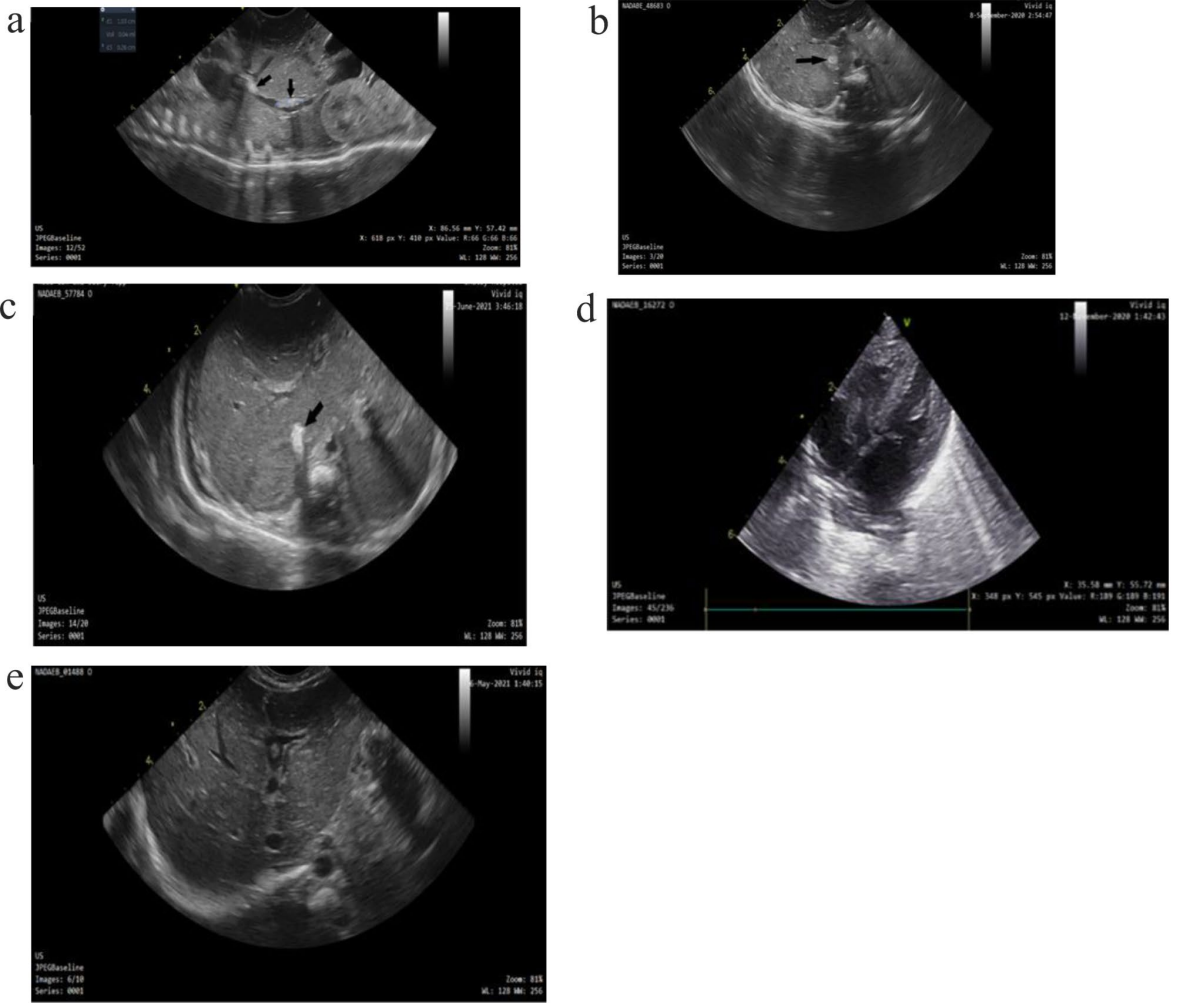
Patients having IVC (2a,2b,2c), cardiac (2d), and umbilico-portal confluence (2e). **a** Shows US images of a neonate with GA of 33 weeks and with femoral catheter after 20 days of catheter insertion. Sagittal view shows two hyperechoic thrombi in intrahepatic IVC (black arrows). The largest one is at the tip of the catheter measuring 10.3 in length and 2.6 mm in thickness. **b** Shows US images of another neonate with GA of 31 weeks with inserted femoral catheter. The axial view shows a hyperechoic thrombus (black arrow) around the catheter in intrahepatic portion of IVC, totally occluding its lumen. **c** Shows US images of a neonate with GA of 29 weeks and with femoral catheter after 10 days of catheter insertion. Axial view shows increase in size of the IVC thrombus on follow up examination (black arrow). **d** Shows different US images of a neonate with GA of 34 week with thrombosis diagnosed after removal of UVC. On follow up examination, increase in size of the thrombus is noted and the thrombus extended through the tricuspid valve. **e** Shows US images of a neonate with GA of 40 weeks and with inserted UVC after 4 days of catheter insertion. Axial view shows the thrombus in umbilico-portal confluence

During the research time from July 2020 to July 2021, 147 neonates were eligible for investigation as they needed CVCs insertion immediately after admission or during their hospital stay in the NICU. Out of those147 patients, thirteen neonates did not complete the study as they died before 7 days of age. This was considered as sample loss, and then 134 neonates completed the study. The neonates in our investigation had 142 CVCs, including 95 (66.9%) UVCs, 39 (27.5%) femoral venous catheters, 6 (4.2%) jugular venous catheters, and 2 (1.4%) PICCs. One hundred twenty-seven neonates received 1 CVC, and the remaining neonates received 2 (*n* = 6), or 3 (*n* = 1) CVCs (Fig. 1). A total of 17 cases (12%) of catheters thrombosis were found during the placement of 142 catheters or 1615 catheter days, which resulted in an overall rate of 10.5 thrombotic events per 1000 catheter days. Figure 2 shows imaging of 5/17 patients who had developed CVC-linked thrombosis. While, S-Fig. 1 shows the imaging of all seventeen patients who had the CVC-linked thrombosis in the study period.

Table 1 shows distribution of patients according to demographic and perinatal data. S-Table 1 shows descriptive data of the maternal risk factors and clinical course of patients enrolled in the study during their NICU stay. S-Table 2 shows description of thrombi discovered at each scan regarding site, thickness, length, echogenicity, and state of venous occlusion. Also shows descriptive analysis for vital signs and laboratory data during examination. In the whole sample, catheter dwell time ranged from 1 to 36 days, with a mean of 11.4 ± 5.3, and median IQR 11.0 (8.0–14.0). The highest rate of thrombosis was associated with femoral NT-CVL with 28.2% of the patients having same insertion site, Table 2. In UVC, femoral CVC, and jugular CVC, median thrombus development times were 9, 12, and 9 days, respectively. The highest rates (72.7%) of thrombus progression were with femoral NT-CVL. In the current work, 10/17 patients with thrombosis received therapeutic doses of enoxaparin sodium, low molecular weight heparin, whilst 7/17 patients did not receive any anticoagulant medication due to presence of thrombocytopenia (< 50 × 10^3^ cell/dl) and/or bleeding.

**Table 1:** Distribution of the studied cases according to demographic data and perinatal data (*n* = 134)

		No.	%
**Sex**	Male	64	48%
	Female	70	52%
**Gestational age (weeks)**			
Min. – Max.		27.00–41.00	
Mean ± SD.		32.91 ± 3.40	
Median (IQR)		32.00 (30.00–35.00)	
**Birth weight (grams)**			
Min. – Max.		610.0–4700.0	
Mean ± SD.		1715.36 ± 902.5	
Median (IQR)		1400 (1100–2000)	
**Mode of delivery**	NVD	35	26%
	CS	99	74%
**Resuscitation**	Routine care	3	2.2%
	Initial steps	89	66.4%
	PPV & mask	30	22.4%
	ETT	11	8.2%
	ETT & Chest compression	1	0.7%
**Meconium**	No	125	88%
	Meconium vigorous	2	1.4%
	Meconium flaccid	7	4.9%
**APGAR score at 1 min**			
Min. – Max.		1.00–7.00	
Mean ± SD.		5.50 ± 1.11	
Median (IQR)		6.00 (5.00–6.00)	
**APGAR score at 5 min**			
Min. – Max.		4.00–9.00	
Mean ± SD.		7.91 ± 0.92	
Median (IQR)		8.00 (7.00–9.00)	

IQR: Inter quartile range SD: Standard deviation LL: Lower limit UL: Upper Limit Cs: C- section NVD: normal vaginal delivery PPV: positive pressure ventilation ETT: endotracheal tube

**Table 2:** Distribution of the thrombus as regards the central venous catheter (*n* = 142)

		Central venous catheter			
		UVC	Femoral	Jugular	PICC
**Thrombus**	No	90 (94.7%)	28 (71.8%)	5 (83.3%)	2 (100.0%)
	Yes	5 (5.3%)	11 (28.2%)	1 (16.7%)	0 (0.0%)
**Thrombus on which exam**	First	1 (20.0%)	0 (0.0%)	0 (0.0%)	0 (0.0%)
	Second	2 (40.0%)	6 (54.5%)	1 (100.0%)	0 (0.0%)
	Third	0 (0.0%)	1 (9.1%)	0 (0.0%)	0 (0.0%)
	Fourth	0 (0.0%)	3 (27.3%)	0 (0.0%)	0 (0.0%)
	After removal	2 (40.0%)	1 (9.1%)	0 (0.0%)	0 (0.0%)
**Thrombus on which catheter days**					
Min. – Max.		3.0–14	5.0–25.0	9.0–9.0	.
Mean		10.2	13.7	9.0	.
Median		9.0	12.0	9.0	.
**Thrombus on which postnatal age (days)**					
Min. – Max.		4.0–16	12.0–38.0	61.0–61.0	.
Mean		10.2	26.1	61.0	.
Median		9.0	27.0	61.0	.
**Fate of thrombus**	Resolved	3 (60.0%)	3 (27.3%)	1 (100.0%)	0 (0.0%)
	Unresolved & patients ‘death	2 (40.0%)	8 (72.7%)	0 (0.0%)	0 (0.0%)

IQR: Inter quartile range SD: Standard deviation LL: Lower limit UL: Upper Limit

S-Table 2 shows the site, echogenicity, length, and thickness of thrombi and laboratory investigations in each examination. The majority of thrombi in different examinations were isoechoic. The longest and thickest thrombi were found on the third examination. The type of central line affected the location of thrombi. Out of the five patients diagnosed with UVC-linked thrombosis, three experienced thrombi at umbilico-portal confluences, and two experienced intracardiac thrombi. Among eleven patients with femoral CVL, one patient had IVC thrombi with common femoral and superficial femoral extensions, and two patients had cardiac extensions. A cardiac thrombus was found in one patient with jugular CVL. We constructed a logistic regression model to identify risk factors behind CVC-linked thrombosis. In univariate analysis, femoral NT-CVL, catheter dwell-time, intraventricular hemorrhage, sepsis, as well as PRBCs transfusions and low platelet count were risk factors for CVC-linked thrombosis, S-Table 3. Nevertheless, the multivariate analysis revealed that only blood transfusion was significant, Table 3, with OR and 95% confidence level 5.768 (1.013–32.836). Duration of hospital stay ranged from 4 to 100 days, with mean value 31.4 ± 21.2 days, and was similar in thrombotic and non-thrombotic group. Deaths were higher in thrombotic group with P value = 0.012 (S-Table 3). The fate/outcome of thrombi was demonstrated in Fig. 1.

**Table 3:** Multivariate logistic regression analysis for the most independent factors affecting thrombus development (*n* = 142)

	P value	OR	Multivariate analysis	
			95% C.I. for OR	
			Lower	Upper
Catheter dwell time	0.395	1.047	0.942	1.162
Central line type (femoral)	0.164	2.521	0.686	9.262
PRBCS transfusion (yes)	0.048	5.768	1.013	32.836
Sepsis (yes)	0.918	0.936	0.264	3.323
Platelet count	0.073	0.995	0.989	1.000

OR: Odd`s ratio

C.I: Confidence interval LL: Lower limit UL: Upper Limit

#: All clinical and investigational variables with *p* < 0.05 in Univariate was included in the multivariate

*: Statistically significant at *p* ≤ 0.05

**Fig. 3:**
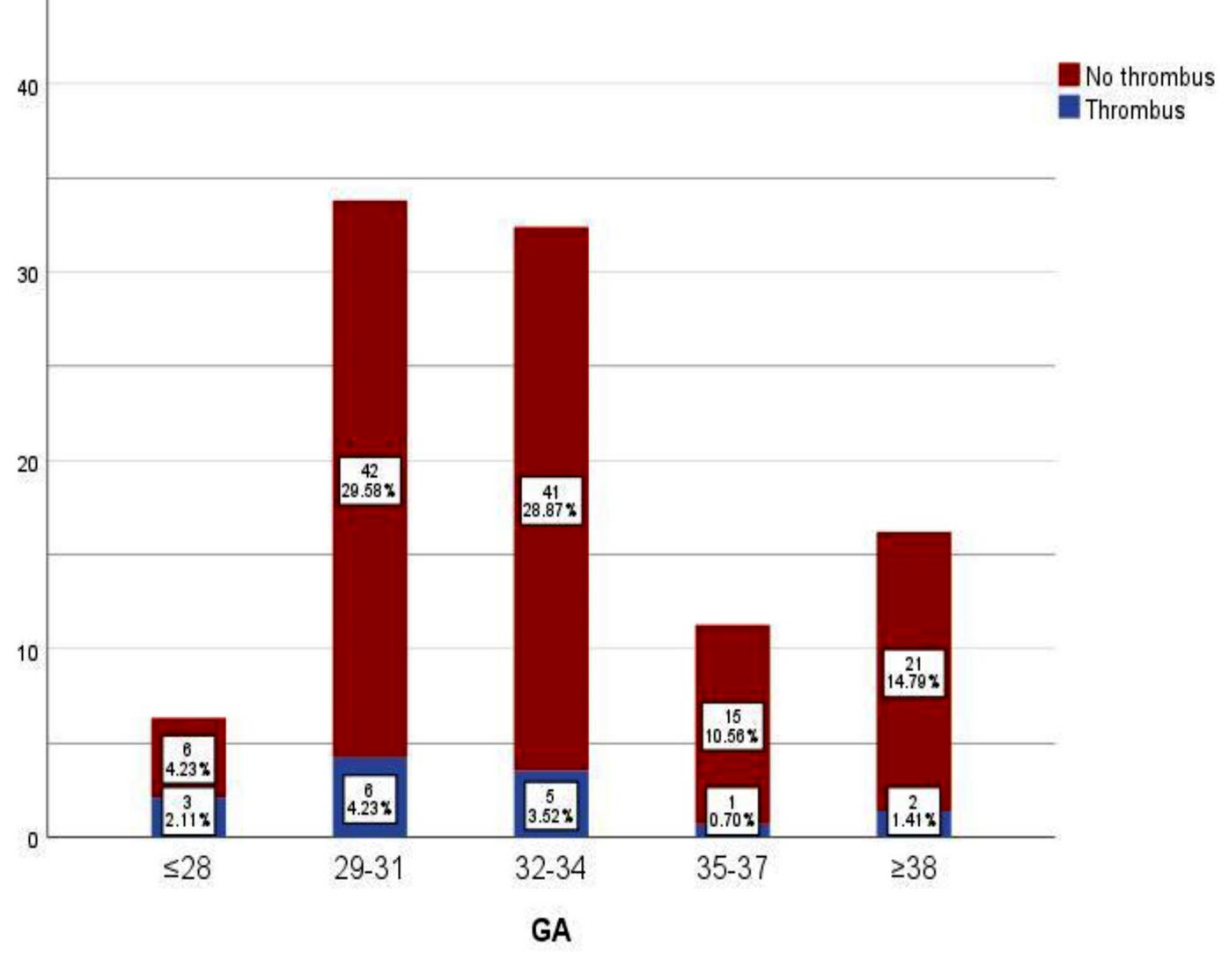
The diagram showed that thrombosis incidence varies based on gestational age. Most catheter-related thromboses occured in the 29-31 week gestational age range, followed by 32-34 week gestational age range

Figure 3 demonstrates that the incidence of thrombosis changes with gestational age. The 29–31week gestational age range exhibited the highest incidence of CVCs-linked thromboses, followed by the 32–34week gestational age range.

## Discussion

There are multiple studies in the literature discussing CVC-linked thrombosis. However, more data are needed especially from developing countries about magnitude of problem and risk factors related to CVC-linked thrombosis. The incidence of CVCs-related thrombosis in infants and children varies from 1.3 to 67% ^(6)^.

A prospective study, conducted by Kim et al. in 2001 [6] on 100 neonates with UVCs, reported portal venous thrombosis development in a large number (43%) of infants. The risk of thrombosis was significantly increased if catheter indwelling days were > 6 (P.001), blood products were added to the UVC-infusate (P-value 0.019) and if catheter tip was present in the liver.

Another study was conducted by Gharehbaghi et al. in 2011 [7], at Al Zahra hospital, Tabriz, Iran on 164 neonates who had UVC and/or UAC insertion. They reported low incidence of catheter related portal vein thrombosis (5/164 patients). However, the duration of catheter dwell time in the study was low in comparison to the other similar studies, with mean value 3.4 ± 1.94 days. In the current work, the mean CVCs’ dwell time was 11 days in patients with no thrombosis in comparison to 15 days in patients with thrombosis, p value 0.005.

Hwang et al.,2020 [8] studied 137 neonates for umbilical catheters-linked thrombosis in the NICU of Haeundae Paik Hospital, Republic of Korea. They reported 19% incidence of catheter-linked thrombosis in UVC and UAC, and that high serum calcium concentration was a significant risk factor (95% confidence interval, 1.26–15.32; *P* = 0.020).

The first study comparing the incidences of thrombosis in 3 of the most widely used CVC types in a large cohort of neonates was the study conducted by Dubbink-Verheij et al., 2017 [9], at the NICU of Leiden University Medical Center, Netherlands. However, the study was of retrospective design and ultrasound of the catheter was not performed routinely in all neonates with catheters, but only when symptoms of thrombi were present. Therefore, of a total of 552 neonates receiving 656 catheters, Dubbink-Verheij et al. detected thrombosis in only 14 patients, with overall incidence of 2.1% or 3.6 thrombotic events/1000 catheter days. [9]

Identifying risk factors for catheter-linked thrombosis might be crucial in prevention, prediction and early treatment. Indwelling catheter is regarded as the single most significant risk factor for thrombosis, however other risk factors also might be involved.

Genes and epigenetic regulation of genes through DNA methylation play a growing role in diagnosis of neonatal diseases [10–12]. Acquired and inherited prothrombotic risk factors increase the risk of thrombosis in neonates. There are 3 main genes whose mutations might play a major role in neonatal hereditary thrombophilia. They are the factor-V-Leiden mutation, methylenetetrahydrofolate reductase (*MTHFR*) mutation, and the prothrombin gene mutation. However, there is no solid data about their relation with catheter related thrombosis. Giuffrè et al. reported association between a catheter-related portal vein thrombosis in a preterm newborn affected with fungal infection and mutation of the *MTHFR* and plasminogen activator inhibitor-1 (*PAI-1*) genes. [13] CVC in neonate is considered one of major acquired prothrombotic risk factors. Other reported risk factors are different and of variable importance in the published studies [6–9].

In the current work, we constructed a regression model to identify risk factors that might have played a role in developing catheter-linked thrombi. In the univariate analysis we reported that sepsis, catheter dwell-time, femoral NT-CVL, low platelet count and PRBCs transfusions were risk factors for catheter- linked thrombosis, S- Table 3. However, only PRBCs transfusion was significant in the multivariate analysis, Table 3, with OR and 95% confidence level of 5.768 (1.013–32.836).

There is a strong relation between sepsis and catheter-linked thrombosis. [13, 14] Infection promotes clotting activation and catheters provide a center for thrombus formation. Thornburg et al. identified 142 cases of CLABSI among 212 PICCs removed because of thrombosis [14].

The duration of which the central lines were in situ was also associated with thrombosis, and the use of CVCs is thus recommended to be as short as possible [15]. To avoid infection, some researchers advise taking out the UVC no more than 14 days after it is placed [3]. According to the current guidelines by standards of infusion therapy, UVCs dwell time should be limited to 7 to 10 days, to reduce risks of infectious and thrombotic complications and to be replaced by PICC lines up to 28 days [16]. Prolonged parenteral nutrition is often mandatory for postoperative patients, as well as for patients with growth restrictions and certain congenital defects [17, 19].

However, according to our results, thrombosis was diagnosed in the second scan in 10/17 patients at 7–10 days after catheter insertion. Median time for UVCs- related thrombosis was 9 days, and femoral CVLs was 12 days. Therefore, we would recommend that catheter dwell time should not exceed 9 days. In situations where the clinicians would keep CVCs more than 7–10 days in UVCs and 12 days in femoral CVLs, ultrasound scan would be recommended. Nevertheless, it was noticed that thrombosis in UVC and femoral NT-CVL occurred as early as 3rd and 5th days after insertion in two of the studied patients, respectively (Table 2).

In the current study, we found that femoral CVL is risk factor for catheter-linked thrombosis with 28.2% (11/39), followed by UVC with 5.36% (5/95). Dubbink-Verheij et al. similarly found that the incidence of thrombosis was higher in femoral NT-CVLs, 7.8% (5/64) than other CVCs, followed by UVC with 1.7% (7/407). The insertion of UVC through necrotic tissue and femoral NT-CVL insertion through relatively unclean area might be the factors behind development of early thrombosis.

In the current study, the platelet count was significantly less in the thrombosis group with mean 152.6 ± 200.8, median 120.0 (23.0–170.0) Vs mean 220.1 ± 89.7 and median 206.0 (160.0–273.0) in the No-thrombosis group, with P value = 0.014. Thrombocytopenia was significantly higher in the thrombosis group (58.8%) than in the No thrombosis group (28.0%) with P value = 0.014. This can be explained by increased platelet consumption in the thrombus. However, in the study of Dubbink-Verheij et al. [8], thrombocytopenia was not statistically different between the different catheter groups and between neonates with and without thrombi.

Neonates who received PRBCs through PIVs at least once during hospital stay were at a much higher rate of thrombosis; 88.2% received PRBCs through PIVs at least once during hospital stay in thrombotic group versus 36.8% of the non-thrombotic group. Kim et al. [5] found that blood products added to umbilical venous catheter infusions significantly increased the chance of clot formation in 47 babies (P value = 0.019). The increased viscosity of the blood following PRBCs-transfusion may increase the risk of thrombosis. Blood transfusions may cause hypercoagulability due to their proinflammatory and immunomodulatory properties. Thrombotic complications were associated with transfusion of blood products in neonates and young infants undergoing cardiac surgery in a study by Faraoni et al. [20]. According to Narag et al., the most significant risk factor associated with CVC-linked thrombosis in very low birth weight infants was a high HCT level [21].

Blood transfusion also increases the risk of thrombosis in adults, according to various studies in the literature. In hospitalized cancer patients, transfusion of both RBCs and platelets increased the risk of thromboembolism [22]. Blood transfusion as a risk factor for venous thromboembolism was reported by Gangireddy et al. [23] and Khorana et al. [24]. They found that both PRBC and platelet transfusions were associated with increased venous and arterial thrombotic events and mortality in cancer patients.

Finally, despite gestational age was not reported as a risk factor for catheter-linked thrombosis in the logistic regression model of the current study, we found that the incidence of thrombosis varied based on gestational age. Most of catheter-linked thromboses occurred in the 29–31-week gestational age range, (Fig. 3).

### Limitation of study

We lacked long-term follow-up of patients to detect the development of portal hypertension. None of our patients had portal vein thrombosis, while three patients had umbilico-portal confluence thrombosis.

Additionally, the current study did not investigate the role of congenital prothrombotic disorders in CVC-linked thrombosis.

Not all inserted CVCs were ultrasound guided. Nevertheless, the insertion of ultrasound-guided CVCs reduces primarily mechanical complications during insertion and is not directly related to thrombus formation in neonates.

Patients with thrombosis were not treated with thrombolytics or surgical thrombectomy because they were critically ill and could not tolerate either procedure. Additionally, patients had no clinically detected local ischemic manifestations.

A further limitation was that catheters were not immediately removed once thrombosis was detected, as some authors recommend, but there are no established guidelines for treating neonates with CVC-related thrombosis.

Because we had a separate surgical NICU in our hospital, patients with surgical indications for CVCs were not included in the study.

## Conclusions

Despite the CVC is the life line of the critically ill neonates, CVC-linked thrombosis is a major threat that can adversely affect patient’s morbidity and mortality. According to the current study, catheter-linked thrombosis occurred in 12% of patients, with an overall rate of 10.5 events per 1000 catheters’ days.

Many factors should be considered in prediction of patients at risk of catheter-linked thrombosis in neonates including: sepsis, catheter dwell time, femoral CVLs insertion, low platelet count and PRBCs- transfusion even when infused through separate PIVs. In our analysis, PRBCs-transfusion was the most powerful factor associated with CVC-linked thrombosis.

The Doppler scan should target the anatomical locations at which catheters are likely to develop thrombi, such as the umbilico-portal confluence and the right side of the heart in UVCs, the IVC in femoral CVLs, and the right side of heart in jugular CVLs. Additionally, the scans may be recommended for patients whose catheter dwell time exceeds 9th day and 12th day for UVCs and femoral CVLs, respectively.

### Electronic supplementary material

Below is the link to the electronic supplementary material.

Supplementary Material 1

Supplementary Material 2

Supplementary Material 3

Supplementary Material 4

## Data Availability

The datasets generated during and/or analyzed during the current study are available from the corresponding author on reasonable request.

## Abbreviations

UVC: Umbilical vein catheters
CVC: Central venous catheters
CL: Central lines
NT-CVL: Non tunneled central venous lines
IVC: Inferior vena cava
SVC: Superior vena cava
RA: Right atrium
LA: Left atrium
RV: Right ventricle
PIVs: Peripheral intravenous lines
PICC: Peripherally inserted central catheter
BSI: Blood stream infection
PRBCs: Packed red blood cells
