# Supplementary material for: Catheters linked thrombosis in neonates: a single center observational study
Source: Ital J Pediatr. 2024 Aug 13;50:147. doi: 10.1186/s13052-024-01708-8 (PMC11320773; doi:10.1186/s13052-024-01708-8)
Supplement: Supplementary file 1 — Supplementary Material 1 [file 13052_2024_1708_MOESM1_ESM.docx]

**S-Table (2a): Descriptive analysis of the studied cases as regards thrombus characteristics and vital signs in the first examination (n = 142)**

|  | | **No.** | **%** |
| --- | --- | --- | --- |
| **Thrombus** | No | 141 | 99.3% |
|  | Yes | 1 | 0.7 % |
| **Site (1)** | IVC | 0 | 0.0% |
|  | Intracardiac | 0 | 0.0% |
|  | Umbilicoportal confluence | 1 | 100.0% |
|  | Portal vein | 0 | 0.0% |
|  | IVC with heart extension | 0 | 0.0% |
|  | IVC with renal veins extension | 0 | 0.0% |
|  | Common femoral vein | 0 | 0.0% |
| **Length (mm) (1)** | |  | |
| Min. – Max. | | 4.0 – 4.0 | |
| Mean ± SD. | | 4.0 | |
| Median (IQR) | | 4.0 | |
| **Thickness (mm) (1)** | |  | |
| Min. – Max. | | 2.0 – 2.0 | |
| Mean ± SD. | | 2.0 | |
| Median (IQR) | | 2.0 | |
| **Age (1)** | Anechoic | 0 | 0.0% |
|  | Hypoechoic | 0 | 0.0% |
|  | Isoechoic | 1 | 100.0% |
|  | Hyperechoic | 0 | 0.0% |
| **Lumen occlusion (1)** | Partial | 1 | 100.0% |
|  | Total | 0 | 0.0% |
| **(Vital signs)** | |  | |
| **HR (b/min)** | |  | |
| Min. – Max. | | 100.0 – 190.0 | |
| Mean ± SD. | | 147.5 ± 17.5 | |
| Median (IQR) | | 150.0 (140.0 – 160.0) | |
| **SPO2 (%)** | |  | |
| Min. – Max. | | 91.0 – 100.0 | |
| Mean ± SD. | | 97.2 ± 1.8 | |
| Median (IQR) | | 98.0 (97.0 – 98.0) | |
| **SBP (mmHg)** | |  | |
| Min. – Max. | | 36.0 – 106.0 | |
| Mean ± SD. | | 65.0 ± 13.2 | |
| Median (IQR) | | 64.0 (54.0 – 75.0) | |
| **DBP (mmHg)** | |  | |
| Min. – Max. | | 14.0 – 70.0 | |
| Mean ± SD. | | 33.6 ± 9.8 | |
| Median (IQR) | | 32.0 (28.0 – 37.0) | |
| **MAP (mmHg)** | |  | |
| Min. – Max. | | 23.0 – 83.0 | |
| Mean ± SD. | | 43.5 ± 10.7 | |
| Median (IQR) | | 44.0 (36.0 – 49.0) | |
| **HCT (%)** | |  | |
| Min. – Max. | | 19.8 – 61.0 | |
| Mean ± SD. | | 40.8 ± 8.9 | |
| Median (IQR) | | 41.0 (34.5 – 47.4) | |
| **WBC (×10^3^/µL)** | |  | |
| Min. – Max. | | 2.8 – 56.0 | |
| Mean ± SD. | | 14.7 ± 9.5 | |
| Median (IQR) | | 12.1 (8.3 – 18.6) | |
| **PLT (×10^3^/µL)** | |  | |
| Min. – Max. | | 15.0 – 559.0 | |
| Mean ± SD. | | 219.9 ± 91.4 | |
| Median (IQR) | | 206.0 (160.0 – 277.0) | |
| **Na (mEq/L)** | |  | |
| Min. – Max. | | 122.0 – 151.0 | |
| Mean ± SD. | | 139.2 ± 5.3 | |
| Median (IQR) | | 139.0 (136.0 – 143.0) | |
| **Ca (mg/dL)** | |  | |
| Min. – Max. | | 6.7 – 12.0 | |
| Mean ± SD. | | 8.8 ± 1.0 | |
| Median (IQR) | | 8.7 (8.2 – 9.3) | |
| **Creatinine (mg/dL)** | |  | |
| Min. – Max. | | 0.1 – 2.6 | |
| Mean ± SD. | | 0.8 ± 0.4 | |
| Median (IQR) | | 0.7 (0.5 – 1.0) | |
| **BUN (mg/dL)** | |  | |
| Min. – Max. | | 5.0 – 52.0 | |
| Mean ± SD. | | 23.0 ± 9.9 | |
| Median (IQR) | | 22.0 (15.0 – 29.0) | |
| **PT (seconds)** | |  | |
| Min. – Max. | | 10.5 – 43.5 | |
| Mean ± SD. | | 15.8 ± 5.1 | |
| Median (IQR) | | 14.6 (12.4 – 17.0) | |
| **PTT (seconds)** | |  | |
| Min. – Max. | | 12.9 – 120.0 | |
| Mean ± SD. | | 54.7 ± 15.1 | |
| Median (IQR) | | 52.0 (46.0 – 60.0) | |

IQR: Inter quartile range SD: Standard deviation LL: Lower limit UL: Upper Limit

HR: Heart rate SPO2: Oxygen saturation SBP: Systolic blood pressure

DBP: Diastolic blood pressure MAP: Mean arterial pressure

**S-Table (2b): Descriptive analysis of the studied cases as regards thrombus characteristics and vital signs in the second examination (n = 101)**

|  | | **No.** | **%** |
| --- | --- | --- | --- |
| **Thrombus** | No | 92 | 91.1% |
|  | Yes | 9 | 8.9% |
| **Site (9)** | IVC | 5 | 55.6% |
|  | Intracardiac | 2 | 22.2% |
|  | Umbilicoportal confluence | 1 | 11.1% |
|  | Portal vein | 0 | 0.0% |
|  | IVC with heart extension | 0 | 0.0% |
|  | IVC with renal veins extension | 0 | 0.0% |
|  | Common femoral vein | 1 | 11.1% |
| **Length (mm) (9)** | |  | |
| Min. – Max. | | 2.5 – 11.0 | |
| Mean ± SD. | | 6.8 ± 2.6 | |
| Median (IQR) | | 6.0 (6.0 – 8.0) | |
| **Thickness (mm) (9)** | |  | |
| Min. – Max. | | 1.5 – 5.0 | |
| Mean ± SD. | | 2.6 ± 1.2 | |
| Median (IQR) | | 2.0 (2.0 – 3.0) | |
| **Age (9)** | Anechoic | 0 | 0.0% |
|  | Hypoechoic | 1 | 11.1% |
|  | Isoechoic | 8 | 88.9% |
|  | Hyperechoic | 0 | 0.0% |
| **Lumen occlusion (9)** | Partial | 8 | 88.9% |
|  | Total | 1 | 11.1% |
| **(Vital signs)** | |  | |
| **HR (b/min)** | |  | |
| Min. – Max. | | 130.0 – 200.0 | |
| Mean ± SD. | | 155.7 ± 14.2 | |
| Median (IQR) | | 153.0 (145.0 – 165.0) | |
| **SPO2 (%)** | |  | |
| Min. – Max. | | 93.0 – 100.0 | |
| Mean ± SD. | | 97.4 ± 1.3 | |
| Median (IQR) | | 98.0 (97.0 – 98.0) | |
| **SBP (mmHg)** | |  | |
| Min. – Max. | | 46.0 – 102.0 | |
| Mean ± SD. | | 69.2 ± 11.1 | |
| Median (IQR) | | 68.0 (62.0 – 75.0) | |
| **DBP (mmHg)** | |  | |
| Min. – Max. | | 20.0 – 78.0 | |
| Mean ± SD. | | 36.2 ± 9.5 | |
| Median (IQR) | | 34.0 (31.0 – 41.0) | |
| **MAP (mmHg)** | |  | |
| Min. – Max. | | 29.0 – 77.0 | |
| Mean ± SD. | | 47.3 ± 9.7 | |
| Median (IQR) | | 45.0 (41.0 – 52.0) | |
| **HCT (%)** | |  | |
| Min. – Max. | | 16.8 – 54.0 | |
| Mean ± SD. | | 35.3 ± 7.8 | |
| Median (IQR) | | 34.2 (30.2 – 41.0) | |
| **WBC (×10^3^/µL)** | |  | |
| Min. – Max. | | 4.3 – 38.9 | |
| Mean ± SD. | | 15.1 ± 7.2 | |
| Median (IQR) | | 14.0 (10.2 – 17.9) | |
| **PLT (×10^3^/µL)** | |  | |
| Min. – Max. | | 13.0 – 808.0 | |
| Mean ± SD. | | 247.7 ± 155.6 | |
| Median (IQR) | | 236.0 (139.0 – 369.0) | |
| **Na (mEq/L)** | |  | |
| Min. – Max. | | 118.0 – 154.0 | |
| Mean ± SD. | | 137.8 ± 5.9 | |
| Median (IQR) | | 137.0 (135.0 – 141.0) | |
| **Ca (mg/dL)** | |  | |
| Min. – Max. | | 7.5 – 12.0 | |
| Mean ± SD. | | 9.1 ± 0.9 | |
| Median (IQR) | | 9.1 (8.5 – 9.6) | |
| **Creatinine (mg/dL)** | |  | |
| Min. – Max. | | 0.1 – 1.9 | |
| Mean ± SD. | | 0.5 ± 0.3 | |
| Median (IQR) | | 0.5 (0.3 – 0.7) | |
| **BUN (mg/dL)** | |  | |
| Min. – Max. | | 2.0 – 58.0 | |
| Mean ± SD. | | 25.8 ± 11.5 | |
| Median (IQR) | | 24.0 (17.0 – 33.0) | |
| **PT (seconds)** | |  | |
| Min. – Max. | | 10.5 – 23.9 | |
| Mean ± SD. | | 14.1 ± 2.0 | |
| Median (IQR) | | 14.0 (12.4 – 15.2) | |
| **PTT (seconds)** | |  | |
| Min. – Max. | | 11.5 – 78.0 | |
| Mean ± SD. | | 49.7 ± 9.8 | |
| Median (IQR) | | 51.0 (44.0 – 55.0) | |

IQR: Inter quartile range SD: Standard deviation LL: Lower limit UL: Upper Limit

HR: Heart rate SPO2: Oxygen saturation SBP: Systolic blood pressure

DBP: Diastolic blood pressure MAP: Mean arterial pressure

**S-Table (2c): Descriptive analysis of the studied cases as regards thrombus characteristics and vital signs in the third examination (n = 22)**

|  | | **No.** | **%** |
| --- | --- | --- | --- |
| **Thrombus** | No | 17 | 77.3% |
|  | Yes | 5 | 22.7% |
| **Site (5)** | IVC | 4 | 80.0% |
|  | Intracardiac | 0 | 0.0% |
|  | Umbilicoportal confluence | 0 | 0.0% |
|  | Portal vein | 0 | 0.0% |
|  | IVC with heart extension | 0 | 0.0% |
|  | IVC with renal veins extension | 0 | 0.0% |
|  | Common femoral vein | 1 | 20.0% |
| **Length (mm) (5)** | |  | |
| Min. – Max. | | 4.0 – 13.0 | |
| Mean ± SD. | | 8.8 ± 3.7 | |
| Median (IQR) | | 8.0 (7.0 – 12.0 ) | |
| **Thickness (mm) (5)** | |  | |
| Min. – Max. | | 2.0 – 4.0 | |
| Mean ± SD. | | 3.0 ± 0.7 | |
| Median (IQR) | | 3.0 (3.0 – 3.0) | |
| **Age (5)** | Anechoic | 0 | 0.0% |
|  | Hypoechoic | 0 | 0.0% |
|  | Isoechoic | 5 | 100.0% |
|  | Hyperechoic | 0 | 0.0% |
| **Lumen occlusion (5)** | Partial | 4 | 80.0% |
|  | Total | 1 | 20.0% |
| **(Vital signs)** | |  | |
| **HR (b/min)** | |  | |
| Min. – Max. | | 125.0 – 190.0 | |
| Mean ± SD. | | 160.1 ± 16.8 | |
| Median (IQR) | | 160.0 (150.0 – 172.0) | |
| **SPO2 (%)** | |  | |
| Min. – Max. | | 94.0 – 100.0 | |
| Mean ± SD. | | 97.1 ± 1.5 | |
| Median (IQR) | | 97.5 (96.0 – 98.0) | |
| **SBP (mmHg)** | |  | |
| Min. – Max. | | 50.0 – 88.0 | |
| Mean ± SD. | | 69.1 ± 9.9 | |
| Median (IQR) | | 69.0 (64.0 – 75.0) | |
| **DBP (mmHg)** | |  | |
| Min. – Max. | | 22.0 – 50.0 | |
| Mean ± SD. | | 34.1 ± 7.0 | |
| Median (IQR) | | 32.5 (30.0 – 40.0) | |
| **MAP (mmHg)** | |  | |
| Min. – Max. | | 35.0 – 62.0 | |
| Mean ± SD. | | 45.4 ± 7.8 | |
| Median (IQR) | | 44.5 (40.0 – 50.0) | |
| **HCT (%)** | |  | |
| Min. – Max. | | 16.7 – 42.3 | |
| Mean ± SD. | | 30.0 ± 7.3 | |
| Median (IQR) | | 29.7 (24.0 – 36.0) | |
| **WBC (×10^3^/µL)** | |  | |
| Min. – Max. | | 3.7 – 29.4 | |
| Mean ± SD. | | 15.2 ± 8.4 | |
| Median (IQR) | | 14.6 (7.5 – 22.8) | |
| **PLT (×10^3^/µL)** | |  | |
| Min. – Max. | | 2.0 – 525.0 | |
| Mean ± SD. | | 167.1 ± 164.8 | |
| Median (IQR) | | 109.0 (50.0 – 205.0) | |
| **Na (mEq/L)** | |  | |
| Min. – Max. | | 131.0 – 147.0 | |
| Mean ± SD. | | 137.4 ± 4.1 | |
| Median (IQR) | | 136.5 (134.0 – 139.0) | |
| **Ca (mg/dL)** | |  | |
| Min. – Max. | | 7.3 – 10.7 | |
| Mean ± SD. | | 8.9 ± 0.9 | |
| Median (IQR) | | 8.8 (8.2 – 9.6) | |
| **Creatinine (mg/dL)** | |  | |
| Min. – Max. | | 0.2 – 1.1 | |
| Mean ± SD. | | 0.5 ± 0.2 | |
| Median (IQR) | | 0.4 (0.3 – 0.5) | |
| **BUN (mg/dL)** | |  | |
| Min. – Max. | | 10.0 – 48.0 | |
| Mean ± SD. | | 21.7 ± 9.8 | |
| Median (IQR) | | 19.0 (15.0 – 26.0) | |
| **PT (seconds)** | |  | |
| Min. – Max. | | 11.4 – 29.5 | |
| Mean ± SD. | | 15.2 ± 3.8 | |
| Median (IQR) | | 14.9 (13.2 – 16.4) | |
| **PTT (seconds)** | |  | |
| Min. – Max. | | 36.2 – 70.0 | |
| Mean ± SD. | | 52.6 ± 10.5 | |
| Median (IQR) | | 52.0 (43.0 – 62.0) | |

IQR: Inter quartile range SD: Standard deviation LL: Lower limit UL: Upper Limit

HR: Heart rate SPO2: Oxygen saturation SBP: Systolic blood pressure

DBP: Diastolic blood pressure MAP: Mean arterial pressure

**S-Table (2d): Descriptive analysis of the studied cases as regards thrombus characteristics and vital signs in the fourth examination (n = 8)**

|  | | **No.** | **%** |
| --- | --- | --- | --- |
| **Thrombus** | No | 3 | 37.5% |
|  | Yes | 5 | 62.5% |
| **Site (5)** | IVC | 2 | 40.0% |
|  | Intracardiac | 0 | 0.0% |
|  | Umbilicoportal confluence | 0 | 0.0% |
|  | Portal vein | 0 | 0.0% |
|  | IVC with heart extension | 2 | 40.0% |
|  | IVC with renal veins extension | 0 | 0.0% |
|  | Common femoral vein | 1 | 20.0% |
| **Length (mm) (5)** | |  | |
| Min. – Max. | | 1.5 – 13.0 | |
| Mean ± SD. | | 6.3 ± 4.9 | |
| Median (IQR) | | 4.0 (3.0 – 10.0) | |
| **Thickness (mm) (5)** | |  | |
| Min. – Max. | | 1.0 – 3.0 | |
| Mean ± SD. | | 1.6 ± 0.9 | |
| Median (IQR) | | 1.0 (1.0 – 2.0) | |
| **Age (5)** | Anechoic | 0 | 0.0% |
|  | Hypoechoic | 1 | 20.0% |
|  | Isoechoic | 4 | 80.0% |
|  | Hyperechoic | 0 | 0.0% |
| **Lumen occlusion (5)** | Partial | 5 | 100.0% |
|  | Total | 0 | 0.0% |
| **(Vital signs)** | |  | |
| **HR (b/min)** | |  | |
| Min. – Max. | | 140.0 – 180.0 | |
| Mean ± SD. | | 160.0 ± 13.1 | |
| Median (IQR) | | 160.0 (150.0 – 170.0) | |
| **SPO2 (%)** | |  | |
| Min. – Max. | | 92.0 – 100.0 | |
| Mean ± SD. | | 96.4 ± 2.9 | |
| Median (IQR) | | 96.0 (94.5 – 99.0) | |
| **SBP (mmHg)** | |  | |
| Min. – Max. | | 58.0 – 88.0 | |
| Mean ± SD. | | 70.5 ± 9.9 | |
| Median (IQR) | | 71.5 (62.5 – 75.0) | |
| **DBP (mmHg)** | |  | |
| Min. – Max. | | 25.0 – 47.0 | |
| Mean ± SD. | | 35.6 ± 9.1 | |
| Median (IQR) | | 34.0 (27.5 – 45.0) | |
| **MAP (mmHg)** | |  | |
| Min. – Max. | | 36.0 – 59.0 | |
| Mean ± SD. | | 46.0 ± 7.8 | |
| Median (IQR) | | 47.5 (39.0 – 50.0) | |
| **HCT (%)** | |  | |
| Min. – Max. | | 18.5 – 36.4 | |
| Mean ± SD. | | 29.5 ± 5.9 | |
| Median (IQR) | | 30.8 (26.1 – 33.8) | |
| **WBC (×10^3^/µL)** | |  | |
| Min. – Max. | | 7.9 – 15.8 | |
| Mean ± SD. | | 12.3 ± 3.0 | |
| Median (IQR) | | 13.4 (9.7 – 14.4) | |
| **PLT (×10^3^/µL)** | |  | |
| Min. – Max. | | 7.0 – 353.0 | |
| Mean ± SD. | | 97.6 ± 117.3 | |
| Median (IQR) | | 50.5 (15.0 – 145.0) | |
| **Na (mEq/L)** | |  | |
| Min. – Max. | | 128.0 – 142.0 | |
| Mean ± SD. | | 137.3 ± 4.3 | |
| Median (IQR) | | 138.0 (136.0 – 140.0) | |
| **Ca (mg/dL)** | |  | |
| Min. – Max. | | 7.4 – 10.7 | |
| Mean ± SD. | | 8.8 ± 1.0 | |
| Median (IQR) | | 9.0 (8.1 – 9.2) | |
| **Creatinine (mg/dL)** | |  | |
| Min. – Max. | | 0.2 – 1.1 | |
| Mean ± SD. | | 0.5 ± 0.3 | |
| Median (IQR) | | 0.4 (0.3 – 0.6) | |
| **BUN (mg/dL)** | |  | |
| Min. – Max. | | 10.0 – 34.0 | |
| Mean ± SD. | | 23.4 ± 7.8 | |
| Median (IQR) | | 24.0 (18.5 – 29.0) | |
| **PT (seconds)** | |  | |
| Min. – Max. | | 11.4 – 22.4 | |
| Mean ± SD. | | 15.3 ± 4.0 | |
| Median (IQR) | | 14.1 (12.0 – 18.1) | |
| **PTT (seconds)** | |  | |
| Min. – Max. | | 36.0 – 164.0 | |
| Mean ± SD. | | 62.1 ± 42.9 | |
| Median (IQR) | | 44.5 (40.5 – 63.5) | |

IQR: Inter quartile range SD: Standard deviation LL: Lower limit UL: Upper Limit

HR: Heart rate SPO2: Oxygen saturation SBP: Systolic blood pressure

DBP: Diastolic blood pressure MAP: Mean arterial pressure

**S-Table (2e): Descriptive analysis of the studied cases as regards thrombus characteristics and vital signs in the after removal examination (n = 113)**

|  | | **No.** | **%** |
| --- | --- | --- | --- |
| **Thrombus** | No | 104 | 92.0% |
|  | Yes | 9 | 8.0% |
| **Site (9)** | IVC | 3 | 33.3% |
|  | Intracardiac | 3 | 33.3% |
|  | Umbilicoportal confluence | 2 | 22.2% |
|  | Portal vein | 0 | 0.0% |
|  | IVC with heart extension | 0 | 0.0% |
|  | IVC with renal veins extension | 0 | 0.0% |
|  | Common femoral vein | 1 | 11.1% |
| **Length (mm) (9)** | |  | |
| Min. – Max. | | 2.2 – 12.0 | |
| Mean ± SD. | | 6.1 ± 3.9 | |
| Median (IQR) | | 4.0 (3.0 – 10.0) | |
| **Thickness (mm) (9)** | |  | |
| Min. – Max. | | 1.0 – 4.0 | |
| Mean ± SD. | | 2.6 ± 0.9 | |
| Median (IQR) | | 3.0 (2.0 – 3.0) | |
| **Age (9)** | Anechoic | 0 | 0.0% |
|  | Hypoechoic | 1 | 11.1% |
|  | Isoechoic | 7 | 77.8% |
|  | Hyperechoic | 1 | 11.1% |
| **Lumen occlusion (9)** | Partial | 8 | 88.9% |
|  | Total | 1 | 11.1% |
| **(Vital signs)** | |  | |
| **HR (b/min)** | |  | |
| Min. – Max. | | 110.0 – 200.0 | |
| Mean ± SD. | | 148.2 ± 15.1 | |
| Median (IQR) | | 145.0 (140.0 – 155.0) | |
| **SPO2 (%)** | |  | |
| Min. – Max. | | 70.0 – 100.0 | |
| Mean ± SD. | | 97.5 ± 2.9 | |
| Median (IQR) | | 98.0 (98.0 – 98.0) | |
| **SBP (mmHg)** | |  | |
| Min. – Max. | | 50.0 – 100.0 | |
| Mean ± SD. | | 68.3 ± 10.9 | |
| Median (IQR) | | 67.0 (62.0 – 75.0) | |
| **DBP (mmHg)** | |  | |
| Min. – Max. | | 21.0 – 65.0 | |
| Mean ± SD. | | 35.8 ± 8.6 | |
| Median (IQR) | | 33.0 (30.0 – 40.0) | |
| **MAP (mmHg)** | |  | |
| Min. – Max. | | 30.0 – 78.0 | |
| Mean ± SD. | | 46.7 ± 9.2 | |
| Median (IQR) | | 46.0 (40.0 – 50.0) | |
| **HCT (%)** | |  | |
| Min. – Max. | | 11.2 – 58.0 | |
| Mean ± SD. | | 36.1 ± 8.8 | |
| Median (IQR) | | 36.6 (30.1 – 41.7) | |
| **WBC (×10^3^/µL)** | |  | |
| Min. – Max. | | 3.3 – 36.0 | |
| Mean ± SD. | | 13.4 ± 6.2 | |
| Median (IQR) | | 11.9 (9.0 – 16.6) | |
| **PLT (×10^3^/µL)** | |  | |
| Min. – Max. | | 21.0 – 1000.0 | |
| Mean ± SD. | | 273.6 ± 172.2 | |
| Median (IQR) | | 241.0 (147.0 – 375.0) | |
| **Na (mEq/L)** | |  | |
| Min. – Max. | | 129.0 – 147.0 | |
| Mean ± SD. | | 139.4 ± 4.2 | |
| Median (IQR) | | 140.0 (136.0 – 142.0) | |
| **Ca (mg/dL)** | |  | |
| Min. – Max. | | 7.0 – 11.6 | |
| Mean ± SD. | | 9.3 ± 0.8 | |
| Median (IQR) | | 9.4 (8.8 – 9.8) | |
| **Creatinine (mg/dL)** | |  | |
| Min. – Max. | | 0.2 – 1.0 | |
| Mean ± SD. | | 0.5 ± 0.2 | |
| Median (IQR) | | 0.5 (0.4 – 0.7) | |
| **BUN (mg/dL)** | |  | |
| Min. – Max. | | 5.0 – 76.0 | |
| Mean ± SD. | | 20.9 ± 11.5 | |
| Median (IQR) | | 19.0 (13.0 – 25.0) | |
| **PT (seconds)** | |  | |
| Min. – Max. | | 10.5 – 17.7 | |
| Mean ± SD. | | 13.5 ± 1.5 | |
| Median (IQR) | | 13.6 (12.0 – 14.6) | |
| **PTT (seconds)** | |  | |
| Min. – Max. | | 14.0 – 64.7 | |
| Mean ± SD. | | 47.6 ± 7.5 | |
| Median (IQR) | | 48.1 (44.0 – 52.0) | |

IQR: Inter quartile range SD: Standard deviation LL: Lower limit UL: Upper Limit

HR: Heart rate SPO2: Oxygen saturation SBP: Systolic blood pressure

DBP: Diastolic blood pressure MAP: Mean arterial pressure
