# Supplementary material for: Catheters linked thrombosis in neonates: a single center observational study
Source: Ital J Pediatr. 2024 Aug 13;50:147. doi: 10.1186/s13052-024-01708-8 (PMC11320773; doi:10.1186/s13052-024-01708-8)
Supplement: Supplementary file 2 — Supplementary Material 2 [file 13052_2024_1708_MOESM2_ESM.docx]

**S- table1:Distribution of patients according to maternal risk factors and their in-hospital courses .**

|  | | **No.** | **%** |
| --- | --- | --- | --- |
| **Antenatal Steroids** | No | 54 | 40.3% |
|  | Incomplete | 37 | 27.6% |
|  | Complete | 43 | 32.1% |
| **Fertilization method** | Spontaneous | 118 | 88.1% |
|  | ICSI | 8 | 6.0% |
|  | Ovulation induction | 6 | 4.5% |
|  | IVF | 2 | 1.5% |
| **Multiplicity** | Single | 97 | 72.4% |
|  | Twin | 36 | 26.9% |
|  | Triplet | 0 | 0.0% |
|  | Quadriplet | 1 | 0.7% |
| **Anaemia** | No | 76 | 56.7% |
|  | Yes | 58 | 43.3% |
| **Vaginitis** | No | 68 | 50.7% |
|  | Yes | 66 | 49.3% |
| **UTI** | No | 82 | 61.2% |
|  | Yes | 52 | 38.8% |
| **PIH** | No | 96 | 71.6% |
|  | Yes | 38 | 28.4% |
| **DM** | No | 129 | 96.3% |
|  | Yes | 5 | 3.7% |
| **Maternal anticoagulants** | No | 122 | 91.0% |
|  | Yes | 12 | 9.0% |
| **Antepartum Hemorrhage** | No | 117 | 87.3% |
|  | Yes | 17 | 12.7% |
| **Maternal fever** | No | 131 | 97.8% |
|  | Yes | 3 | 2.2% |
| **PTLP** | No | 66 | 49.3% |
|  | Yes | 68 | 50.7% |
| **PROM** | No | 107 | 79.9% |
|  | Yes | 27 | 20.1% |

|  | | **No.** | **%** |
| --- | --- | --- | --- |
| **Respiratory support** | No | 15 | 11.2% |
|  | Yes | 119 | 88.8% |
| **Total ventilation days (119)** | |  | |
| Min. – Max. | | 1.0 – 46.0 | |
| Mean ± SD. | | 9.8 ± 9.8 | |
| Median (IQR) | | 6.0 (2.0 – 16.0) | |
| **Inotropes** | No | 79 | 59.0% |
|  | Yes | 55 | 41.0% |
| **Therapeutic Hypothermia** | No | 115 | 85.8% |
|  | Yes | 19 | 14.2% |
| **PPHN** | No | 118 | 88.1% |
|  | on sildenafil | 12 | 9.0% |
|  | on milrinone | 4 | 3.0% |
| **Convulsions** | No | 107 | 79.9% |
|  | Yes | 27 | 20.1% |
| **IVH** | No | 89 | 66.4% |
|  | grade I | 15 | 11.2% |
|  | grade II | 20 | 14.9% |
|  | grade III | 9 | 6.7% |
|  | grade lV | 1 | 0.7% |
| **Hydrocephalus** | No | 126 | 94.0% |
|  | Yes | 8 | 6.0% |
| **PDA** | No | 83 | 61.9% |
|  | Non-significant | 8 | 6.0% |
|  | Significant | 43 | 32.1% |
| **NIHB** | No | 87 | 64.9% |
|  | Phototherapy | 44 | 32.8% |
|  | DVE | 3 | 2.2% |
|  | IVIG | 0 | 0.0% |
| **Pneumothorax** | No | 130 | 97.0% |
|  | Yes | 4 | 3.0% |
| **NEC** | No | 116 | 86.6% |
|  | Yes | 18 | 13.4% |
| **Sepsis** | No | 92 | 64.8% |
|  | Early onset | 27 | 19.0% |
|  | Late onset | 23 | 16.2% |
| **Blood Culture** | Negative | 101 | 71.1% |
|  | Positive | 41 | 28.9% |
| **CLABSI** | Negative | 138 | 97.2% |
|  | Positive | 4 | 2.8% |
| **BAL (24)** | Negative | 2 | 8.3% |
|  | Positive | 22 | 91.7% |
| **Parenteral nutrition** | Partial | 52 | 36.6% |
|  | Total | 90 | 63.4% |
| **PRBCS** | No | 81 | 57.0% |
|  | Yes | 61 | 43.0% |
| **Plasma** | No | 88 | 62.0% |
|  | Yes | 54 | 38.0% |
| **Platelets** | No | 125 | 88.0% |
|  | Yes | 17 | 12.0% |
| **Blood exchange** | No | 136 | 95.8% |
|  | SVE | 3 | 2.1% |
|  | DVE | 3 | 2.1% |
| **Parenteral nutrition** | Partial | 52 | 36.6% |
|  | Total | 90 | 63.4% |
| **PRBCS** | No | 81 | 57.0% |
|  | Yes | 61 | 43.0% |
| **Plasma** | No | 88 | 62.0% |
|  | Yes | 54 | 38.0% |
| **Platelets** | No | 125 | 88.0% |
|  | Yes | 17 | 12.0% |
| **Blood exchange** | No | 136 | 95.8% |
|  | SVE | 3 | 2.1% |
|  | DVE | 3 | 2.1% |
| **Preliminary diagnosis** | RD in preterm | 107 | 79.9% |
|  | RD in full term | 4 | 3.0% |
|  | HIE | 19 | 14.2% |
|  | Jaundice | 2 | 1.5% |
|  | SVT | 1 | 0.7% |
|  | Convulsions | 1 | 0.7% |
| **Hospital stay duration (days)** | |  | |
| Min. – Max. | | 4.0 – 100.0 | |
| Mean ± SD. | | 31.4 ± 21.2 | |
| Median (IQR) | | 27.0 (16.0 – 42.0) | |
| **Outcome** | Discharged | 95 | 70.9% |
|  | Died | 39 | 29.1% |

IQR: Inter quartile range

SD: Standard deviation

LL: Lower limit

UL: Upper Limit

UTI: Urinary tract infection PIH: Pregnancy induced hypertension DM: Diabetes mellitus

PTLP: Preterm labour pain PROM: Prolonged rupture of membrane

PPHN: Persistent pulmonary hypertension

IVH: Intraventricular hemorrhage

PDA: Patent ductus arteriosus

NIHB: Neonatal indirect hyperbilirubinemia

DVE: Double volume exchange

IVIG: Intravenous immunoglobulins

NEC: Necrotizing enterocolitis

CLABSI: Central line associated blood stream infection

BAL: Broncho-alveolar lavage

PICC: Peripherally inserted central catheter

UVC: Umbilical venous catheter

PRBCS: Packed red blood cells

UL: Upper Limit

RD: Respiratory distress

HIE: Hypoxic ischemic encephalopathy

SVT: Supraventricular tachycardia
