# Supplementary material for: Catheters linked thrombosis in neonates: a single center observational study
Source: Ital J Pediatr. 2024 Aug 13;50:147. doi: 10.1186/s13052-024-01708-8 (PMC11320773; doi:10.1186/s13052-024-01708-8)
Supplement: Supplementary file 3 — Supplementary Material 3 [file 13052_2024_1708_MOESM3_ESM.docx]

**S-Table (3a): Univariate analysis for** **the two groups (thrombus and no thrombus) as regards demographic data (n= 142)**

|  | | **No thrombus**  **(n= 125)** | **Thrombus**  **(n= 17)** | **OR (95%C.I)** | **P** |
| --- | --- | --- | --- | --- | --- |
| **Sex** | Male | 60 (48.0%) | 10 (58.8%) | 1.548 (0.554 – 4.325) | 0.405 |
|  | Female | 65 (52.0%) | 7 (41.2%) | 1.000 |  |
| **GA (weeks)** | |  |  |  |  |
| Min. – Max. | | 27.0 – 41.0 | 28.0 – 40.0 | 0.899 (0.762 – 1.062) | 0.210 |
| Mean ± SD. | | 33.1 ± 3.4 | 32.0 ± 3.7 |  |  |
| Median (IQR) | | 32.0 (31.0 – 35.0) | 31.0 (29.0 – 34.0) |  |  |
| **BWT (grams)** | |  |  |  |  |
| Min. – Max. | | 610.0 – 4700.0 | 750.0 – 3600.0 | 0.999 (0.999 – 1.000) | 0.174 |
| Mean ± SD. | | 1765.8 ± 910.4 | 1437.4 ± 908.1 |  |  |
| Median (IQR) | | 1400.0 (1100.0 – 2100.0) | 1020.0 (900.0 – 1350.0) |  |  |
| **SGA** | No | 94 (75.2%) | 10 (58.8%) | 1.000 | 0.159 |
|  | Yes | 31 (24.8%) | 7 (41.2%) | 2.123 (0.744 – 6.052) |  |

IQR: **Inter quartile range** SD: **Standard deviation** OR: **Odd`s ratio**

C.I: Confidence interval LL: Lower limit UL: Upper Limit

p: p value for **Odd`s ratio** for comparing between the studied groups *: Statistically significant at p ≤ 0.05

GA: Gestational age BWT: Birth weight

**S-Table (3b): Univariate analysis for the two groups as regards** **maternal risk factors (n= 142)**

|  | | **No thrombus**  **(n= 125)** | **Thrombus**  **(n= 17)** | **OR (95%C.I)** | **P** |
| --- | --- | --- | --- | --- | --- |
| **UTI** | No | 79 (63.2%) | 9 (52.9%) | 1.000 | 0.416 |
|  | Yes | 46 (36.8%) | 8 (47.1%) | 1.527 (0.551 – 4.231) |  |
| **PIH** | No | 90 (72.0%) | 12 (70.6%) | 1.000 | 0.903 |
|  | Yes | 35 (28.0%) | 5 (29.4%) | 1.071 (0.352 – 3.264) |  |
| **DM** | No | 121 (96.8%) | 16 (94.1%) | 1.000 | 0.579 |
|  | Yes | 4 (3.2%) | 1 (5.9%) | 1.891 (0.199 – 17.982) |  |
| **PROM** | No | 100 (80.0%) | 14 (82.4%) | 1.000 | 0.819 |
|  | Yes | 25 (20.0%) | 3 (17.6%) | 0.857 (0.229 – 3.214) |  |

OR: **Odd`s ratio** p: p value for **Odd`s ratio** for comparing between the studied groups *: Statistically significant at p ≤ 0.05

UTI: Urinary tract infection PIH: Pregnancy induced hypertension DM: Diabetes mellitus

PTLP: Preterm labour pain PROM: Prolonged rupture of membrane

**S-Table (3c): Univariate analysis for the two groups as regards duration of hospital stay and outcome (n= 142)**

|  | | **No thrombus**  **(n= 125)** | **Thrombus**  **(n= 17)** | **OR (95%C.I)** | **P** |
| --- | --- | --- | --- | --- | --- |
| **Hospital stays duration (days)** | |  |  |  |  |
| Min. – Max. | | 5.0 – 100.0 | 4.0 – 95.0 | 1.008 (0.986 – 1.030) | 0.485 |
| Mean ± SD. | | 32.2 ± 21.6 | 36.1 ± 22.6 |  |  |
| Median (IQR) | | 28.0 (16.0 – 42.0) | 35.0 (24.0 – 41.0) |  |  |
| **Outcome** | Discharged | 91 (72.8%) | 7 (41.2%) | 1.000 | 0.012^*^ |
|  | Died | 34 (27.2%) | 10 (58.8%) | 3.824 (1.347 – 10.852) |  |

IQR: **Inter quartile range** SD: **Standard deviation** OR: **Odd`s ratio**

C.I: Confidence interval LL: Lower limit UL: Upper Limit

p: p value for **Odd`s ratio** for comparing between the studied groups *: Statistically significant at p ≤ 0.05

**S-Table (3d): Univariate analysis for the two groups as regards perinatal risk factors (n= 142)**

|  | | **No thrombus**  **(n= 125)** | **Thrombus**  **(n= 17)** | **OR (95%C.I)** | **P** |
| --- | --- | --- | --- | --- | --- |
| **Catheter dwell time (days)** | |  |  |  |  |
| Min. – Max. | | 1.0 – 36.0 | 2.0 – 32.0 | 1.137 (1.039 – 1.243) | 0.005^*^ |
| Mean ± SD. | | 10.9 ± 4.8 | 15.2 ± 7.5 |  |  |
| Median (IQR) | | 11.0 (8.0 – 14.0) | 15.0 (11.0 – 17.0) |  |  |
| **Type** | UVC | 90 (72.0%) | 5 (29.4%) | 0.162 (0.053 – 0.494) | 0.001^*^ |
|  | Femoral | 28 (22.4%) | 11 (64.7%) | 6.351 (2.157 – 18.701) | 0.001^*^ |
|  | Jugular | 5 (4.0%) | 1 (5.9%) | 1.500 (0.165 – 13.667) | 0.719 |
|  | PICC | 2 (1.6%) | 0 (0.0%) | – | 0.999 |
| **Position** | Improper site | 47 (37.6%) | 4 (23.5%) | 1.000 | 0.263 |
|  | Optimal site | 78 (62.4%) | 13 (76.5%) | 1.958 (0.603 – 6.358) |  |
| **PRBCs** | No | 79 (63.2%) | 2 (11.8%) | 1.000 | 0.001^*^ |
|  | Yes | 46 (36.8%) | 15 (88.2%) | 12.880 (2.818 – 58.864) |  |
| **PRBCs/CLABSI** | No | 79 (63.2%) | 2 (11.8%) | 1.000 |  |
|  | With CLABSI | 2 (1.6%) | 1 (5.9%) | 19.750 (1.225 – 318.531) | 0.035^*^ |
|  | Without CLABSI | 44 (35.2%) | 14 (82.4%) | 12.568 (2.730 – 57.855) | 0.001^*^ |
| **PRBCs/Sepsis** | No | 79 (63.2%) | 2 (11.8%) | 1.000 |  |
|  | With Sepsis | 23 (18.4%) | 10 (58.8%) | 17.174 (3.511 – 84.016) | <0.001^*^ |
|  | Without Sepsis | 23 (18.4%) | 5 (28.4%) | 8.587 (1.562 – 47.207) | 0.013^*^ |
| **Plasma** | No | 79 (63.2%) | 9 (52.9%) | 1.000 | 0.416 |
|  | Yes | 46 (36.8%) | 8 (47.1%) | 1.527 (0.551 – 4.231) |  |

|  | | **No thrombus**  **(n= 125)** | **Thrombus**  **(n= 17)** | **OR (95%C.I)** | **P** |
| --- | --- | --- | --- | --- | --- |
| **IVH** | No | 90 (72.0%) | 6 (35.3%) | 1.000 | 0.004^*^ |
|  | Yes | 35 (28.0%) | 11 (64.7%) | 4.714 (1.619 – 13.725) |  |
| **NEC** | No | 108 (86.4%) | 12 (70.6%) | 1.000 | 0.101 |
|  | Yes | 17 (13.6%) | 5 (29.4%) | 2.647 (0.828 – 8.460) |  |
| **Sepsis** | No | 85 (68.0%) | 7 (41.2%) | 1.000 | 0.036^*^ |
|  | Yes | 40 (32.0%) | 10 (58.8%) | 3.036 (1.077 – 8.558) |  |
| **Dehydration** | No | 82 (65.6%) | 9 (52.9%) | 1.000 | 0.311 |
|  | Yes | 43 (34.4%) | 8 (47.1%) | 1.695 (0.610 – 4.707) |  |
| **Thrombocytopenia** | No | 90 (72.0%) | 7 (41.2%) | 1.000 | 0.014^*^ |
|  | Yes | 35 (28.0%) | 10 (58.8%) | 3.673 (1.296 – 10.412) |  |
| **Thrombocytopenia/**  **CLABSI** | No | 90 (72.0%) | 7 (41.2%) | 1.000 |  |
|  | With CLABSI | 2 (1.6%) | 1 (5.9%) | 6.429 (0.517 – 79.950) | 0.148 |
|  | Without CLABSI | 33 (26.4%) | 9 (52.9%) | 3.506 (1.209 – 10.174) | 0.021^*^ |
| **Thrombocytopenia/**  **Sepsis** | No | 90 (72.0%) | 7 (41.2%) | 1.000 |  |
|  | With sepsis | 18 (14.4%) | 8 (47.1%) | 5.714 (1.839 – 17.753) | 0.003^*^ |
|  | Without sepsis | 17 (13.6%) | 2 (11.8%) | 1.513 (0.289 – 7.914) | 0.624 |
|  | |  |  |  |  |
| Min. – Max. | | 15.0 – 559.0 | 7.0 – 808.0 | 0.992 (0.986 – 0.998) | 0.014^*^ |
| Mean ± SD. | | 220.1 ± 89.7 | 152.6 ± 200.8 |  |  |
| Median (IQR) | | 206.0 (160.0 – 273.0) | 120.0 (23.0 – 170.0) |  |  |
| **Calcium (mg/dL)** | |  |  |  |  |
| Min. – Max. | | 6.8 – 12.0 | 7.4 – 9.9 | 0.730 (0.405 – 1.314) | 0.294 |
| Mean ± SD. | | 8.8 ± 1.0 | 8.6 ± 0.7 |  |  |
| Median (IQR) | | 8.7 (8.2 – 9.3) | 8.5 (8.2 – 9.1) |  |  |
| **Leukocytosis** | No | 80 (64.0%) | 11 (64.7%) | 1.000 | 0.955 |
|  | Yes | 45 (36.0%) | 6 (35.3%) | 0.970 (0.336 – 2.798) |  |
| **Polycythemia** | No | 125 (100.0%) | 16 (94.1%) | 1.000 | 0.999 |
|  | Yes | 0 (0.0%) | 1 (5.9%) | – |  |

OR: **Odd`s ratio** p: p value for **Odd`s ratio** for comparing between the studied groups *: Statistically significant at p ≤ 0.05

IVH: Intraventricular hemorrhage NEC: Necrotizing enterocolitis

**S-Table (3e): Univariate analysis for the two groups as regards the inserted central venous catheter and blood products infusion (n= 142)**

|  | | **No thrombus**  **(n= 125)** | **Thrombus**  **(n= 17)** | **OR (95% C.I)** | **P** |
| --- | --- | --- | --- | --- | --- |
| **Catheter dwell time (days)** | |  |  |  |  |
| Min. – Max. | | 1.0 – 36.0 | 2.0 – 32.0 | 1.137 (1.039 – 1.243) | 0.005^*^ |
| Mean ± SD. | | 10.9 ± 4.8 | 15.2 ± 7.5 |  |  |
| Median (IQR) | | 11.0 (8.0 – 14.0) | 15.0 (11.0 – 17.0) |  |  |
| **Type** | UVC | 90 (72.0%) | 5 (29.4%) | 0.162 (0.053 – 0.494) | 0.001^*^ |
|  | Femoral | 28 (22.4%) | 11 (64.7%) | 6.351 (2.157 – 18.701) | 0.001^*^ |
|  | Jugular | 5 (4.0%) | 1 (5.9%) | 1.500 (0.165 – 13.667) | 0.719 |
|  | PICC | 2 (1.6%) | 0 (0.0%) | – | 0.999 |
| **Position** | Improper position | 47 (37.6%) | 4 (23.5%) | 1.000 | 0.263 |
|  | Optimal position | 78 (62.4%) | 13 (76.5%) | 1.958 (0.603 – 6.358) |  |
| **PRBCs** | No | 79 (63.2%) | 2 (11.8%) | 1.000 | 0.001^*^ |
|  | Yes | 46 (36.8%) | 15 (88.2%) | 12.880 (2.818 – 58.864) |  |
| **Plasma** | No | 79 (63.2%) | 9 (52.9%) | 1.000 | 0.416 |
|  | Yes | 46 (36.8%) | 8 (47.1%) | 1.527 (0.551 – 4.231) |  |

IQR: **Inter quartile range** SD: **Standard deviation** OR: **Odd`s ratio**

C.I: Confidence interval LL: Lower limit UL: Upper Limit

p: p value for **Odd`s ratio** for comparing between the studied groups *: Statistically significant at p ≤ 0.05

NB 50 patients had improper position of UVC and 1 patient had improper position of femoral CVC
