# Supplementary material for: Catheters linked thrombosis in neonates: a single center observational study
Source: Ital J Pediatr. 2024 Aug 13;50:147. doi: 10.1186/s13052-024-01708-8 (PMC11320773; doi:10.1186/s13052-024-01708-8)
Supplement: Supplementary file 4 — Supplementary Material 4 [file 13052_2024_1708_MOESM4_ESM.docx]

S-Figure1: Imaging of Seventeen patients having thrombosis from whole study sample.


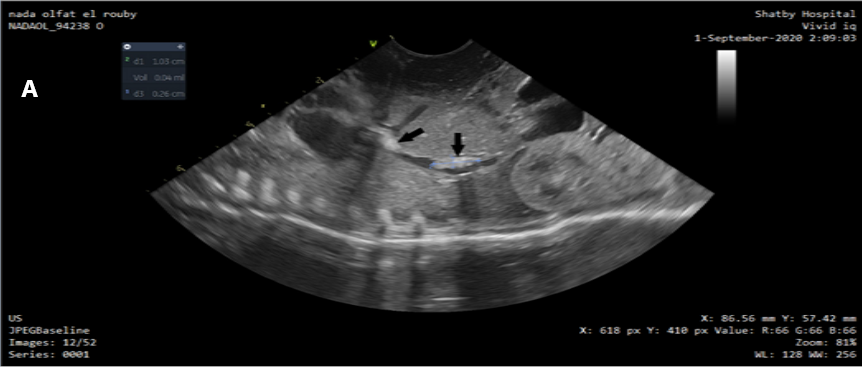


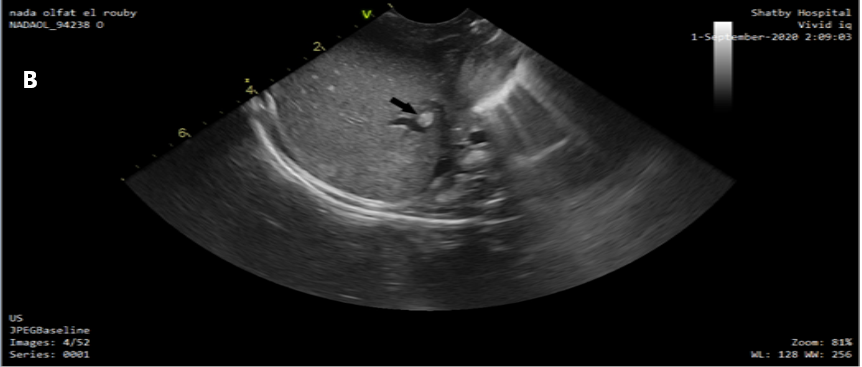


**Patient no.1 imaging:** Shows US images of a neonate with GA of 33 weeks and with femoral catheter after 20 days of catheter insertion. **A.** Sagittal view shows two hyperechoic thrombi in intrahepatic IVC (black arrows) the largest one at the tip of the catheter measuring 10.3 in length and 2.6 mm in thickness. **B.** Axial view of the intrahepatic portion of IVC showing intraluminal large thrombus (black arrow).


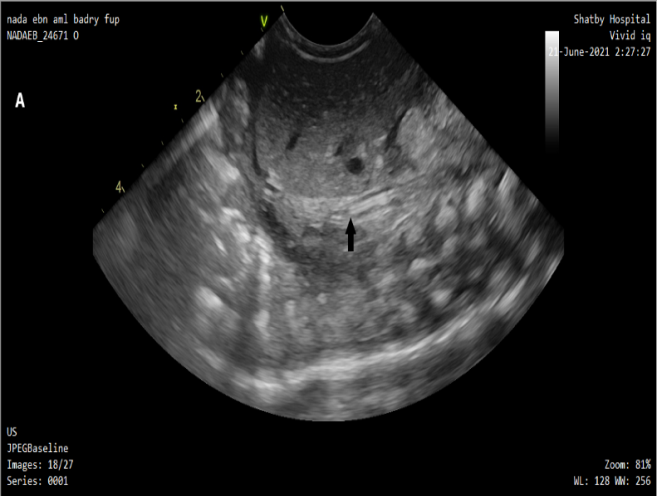

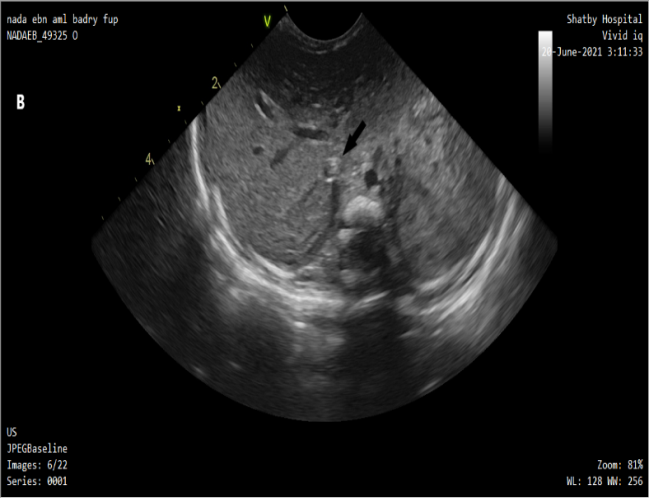


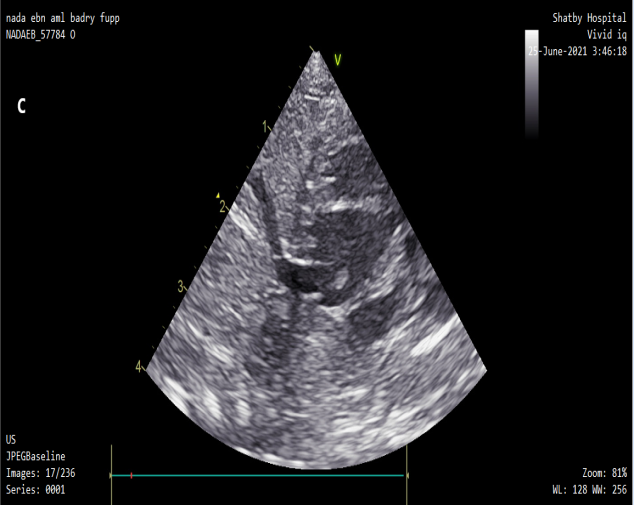

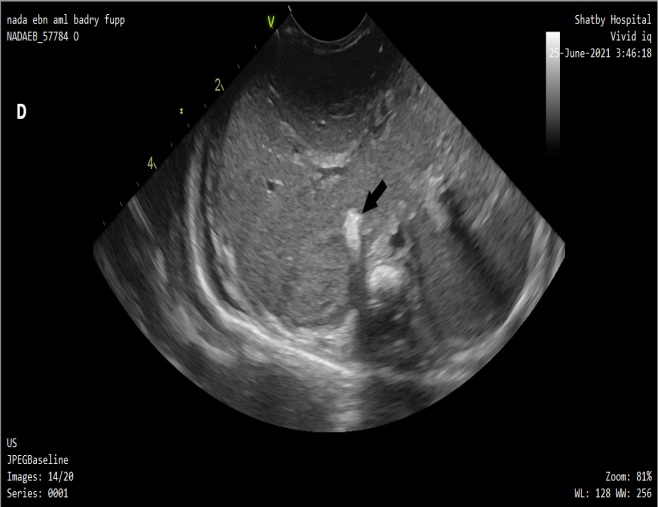


**Patient no. 2 imaging:** Shows US images of a neonate with GA of 29 weeks and with femoral catheter after 10 days of catheter insertion. **A.** Lateral view shows large thrombus in the intrahepatic portion of IVC extending beyond tip of the catheter (black arrow points to the tip of the catheter). **B.** Axial view of the intrahepatic portion of IVC showing intraluminal thrombus (black arrow). **C.** Intracardiac extension of the thrombus on follow up examination. **D.** Axial view shows increase in size of the IVC thrombus on follow up examination (black arrow).


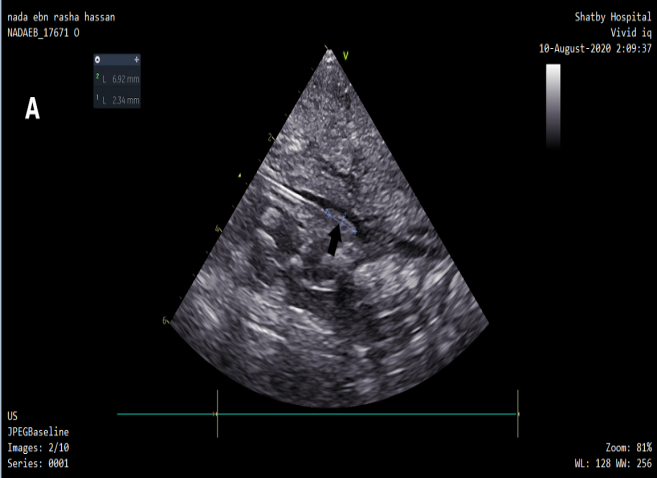

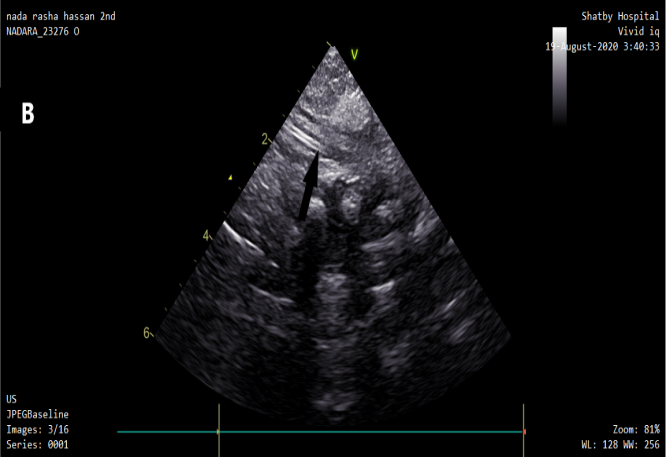


**Patient no.3 imaging:** Shows US images of a neonate with GA of 34 weeks and with inserted femoral catheter. **A.** showing the thrombus at the catheter tip (black arrow) which is 6.9 mm in length and 2.3 mm in thickness. **B.** after resolution of the thrombus after treatment with LMWH.


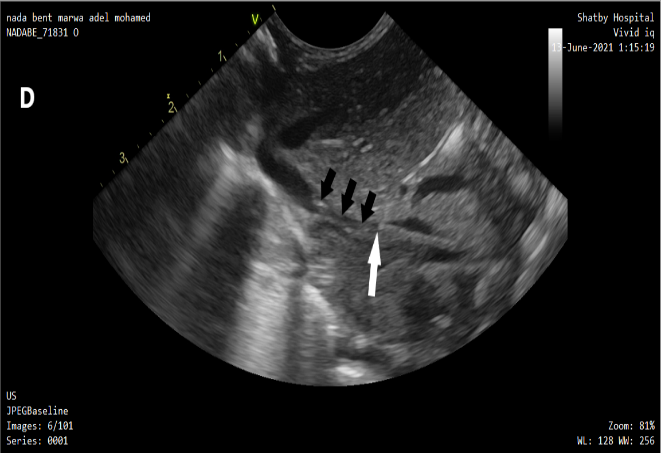

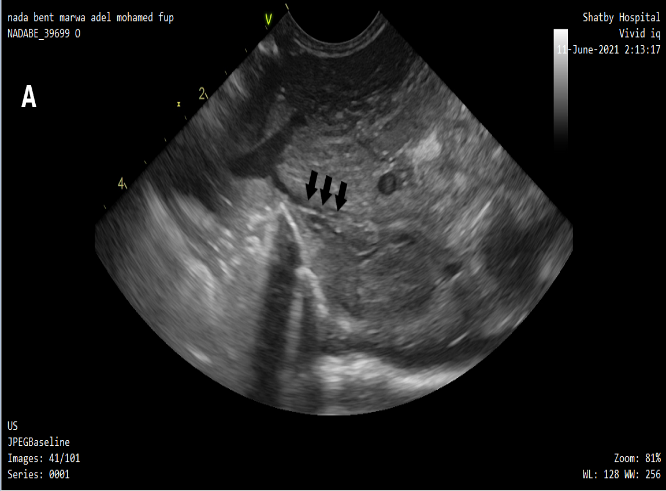

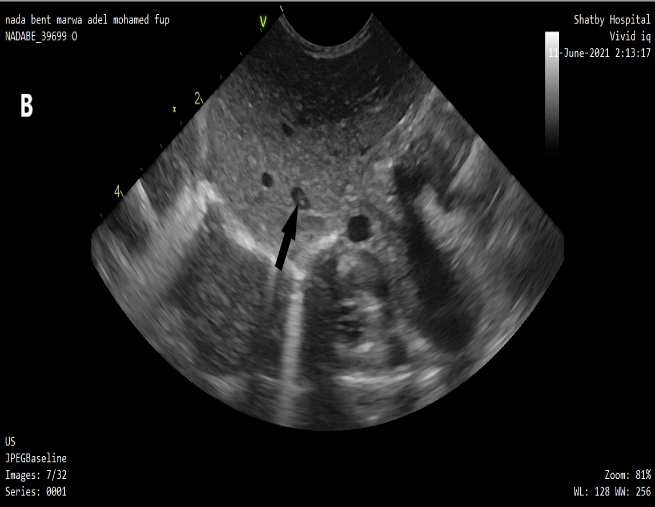


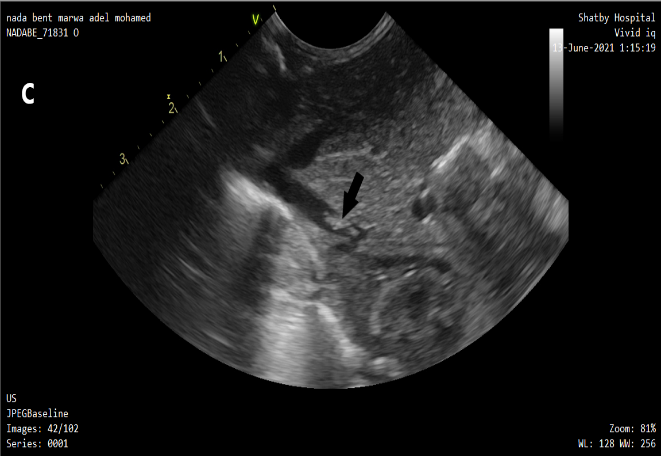


**
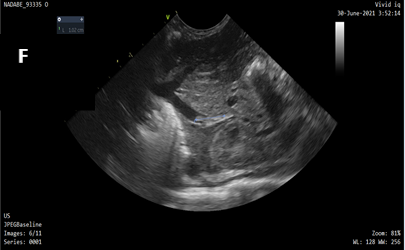
**
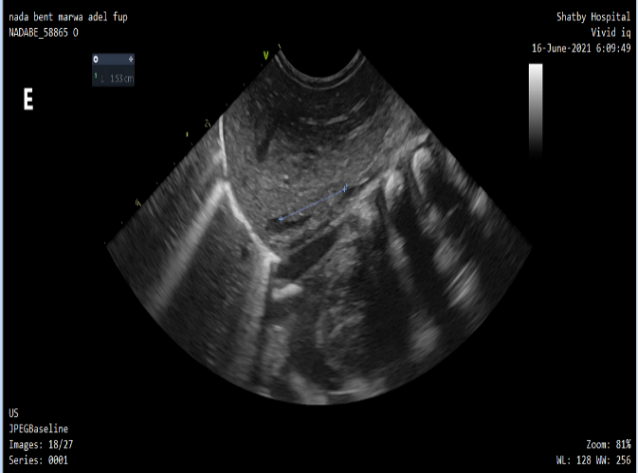


**Patient no.4 imaging:** Shows US images of a neonate with GA of 29 weeks with inserted femoral catheter. **A.** Sagittal view long thrombus (black arrows) in the intrahepatic portion of IVC extending beyond tip of the catheter. **B.** Axial view shows an isoechoic thrombus in the intrahepatic portion of IVC (black arrow). **C. & D.** show increase in size of the thrombus on follow up examination (black arrows point to the thrombus & white arrow points to the tip of the catheter). **E.** Lateral views of the IVC show further increase in size of the thrombus (black arrows) (reaching 1.5 cm in length) despite removal of the femoral catheter. **F.** Sagittal view shows decrease in size of the thrombus after start of treatment with LMWH.


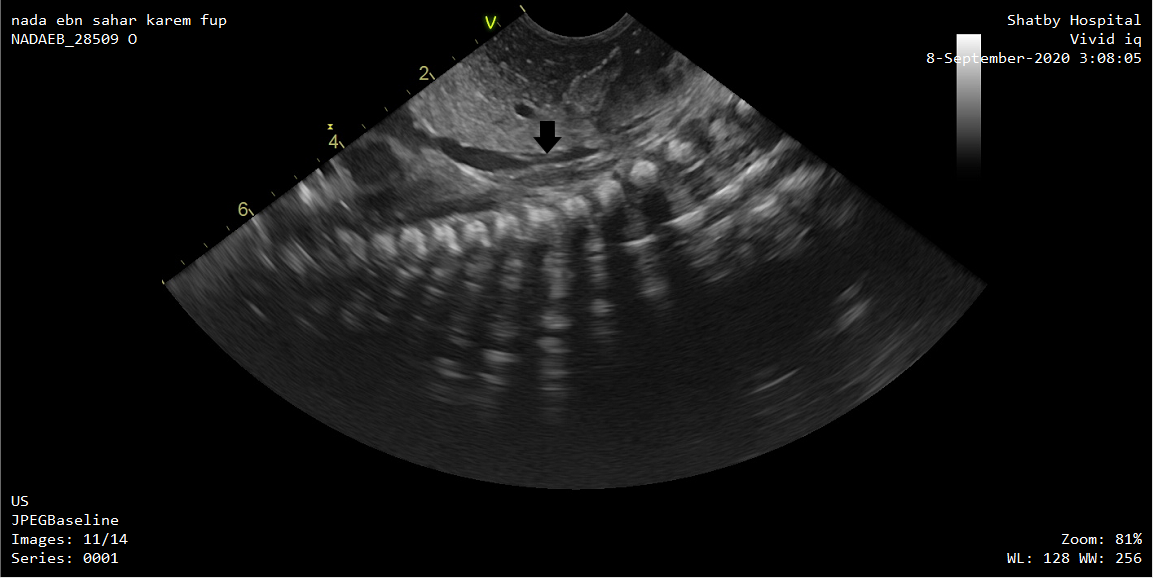


**Patient no.5 imaging:**Shows US image of a neonate with GA of 28 weeks and with femoral catheter after 17 days of catheter insertion. A small long thrombus is noted in IVC (black arrow).


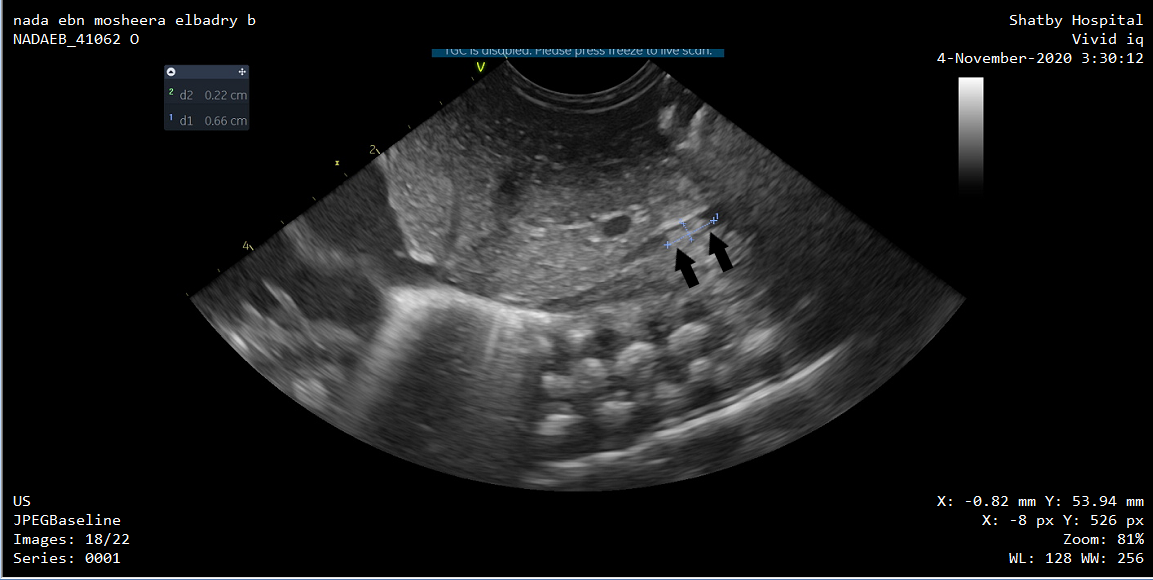


**Patient no.6 imaging:** Shows US image of another neonate with GA of 33 weeks and with femoral catheter after 11 days of catheter insertion. Lateral view shows a thrombus (black arrow) measuring 6.6 mm in length and 2.2 mm in thickness in the intrahepatic portion of IVC.


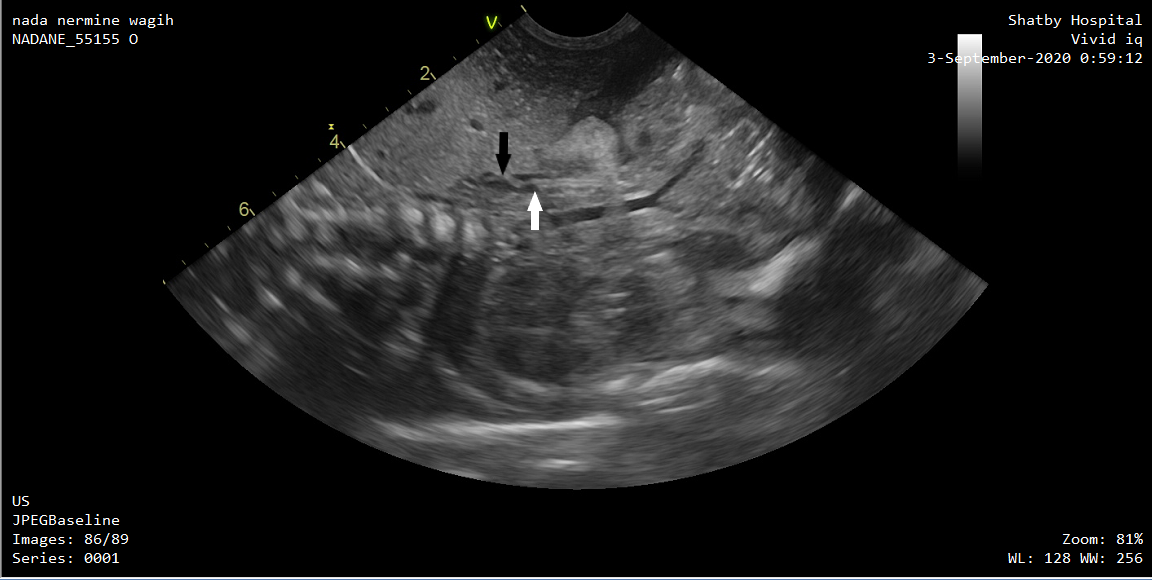


**Patient no.7 imaging:** Shows US image of another neonate with GA of 28 weeks with inserted femoral catheter. The image shows a wavy thrombus (black arrow) attached to the catheter tip (white arrow).


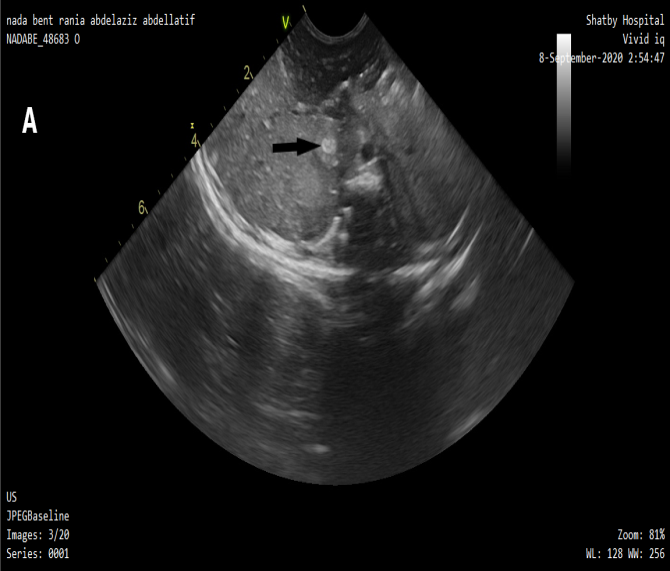

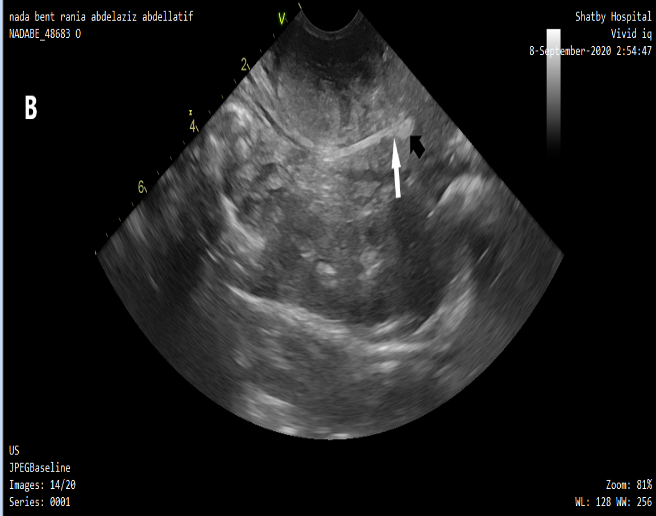


**Patient no.8 imaging:** Shows US images of another neonate with GA of 31 weeks with inserted femoral catheter. **A.** Axial view shows a hyperechoic thrombus (black arrow) around the catheter in intrahepatic portion of IVC totally occluding its lumen. **B.** Lateral view showing the thrombus (black arrow) extending beyond the catheter tip (white arrow).


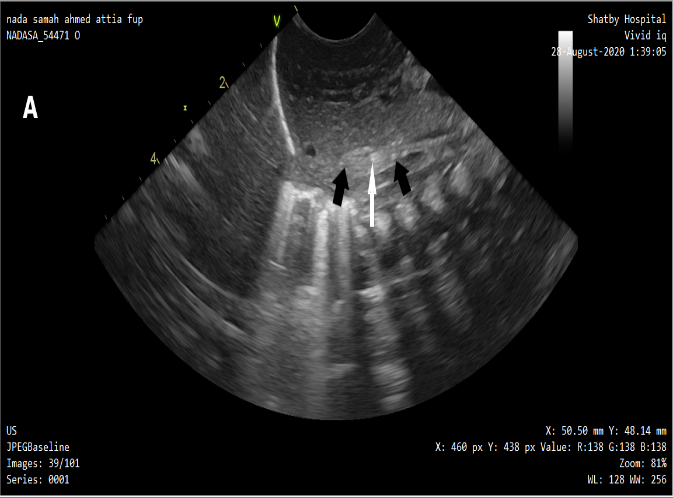

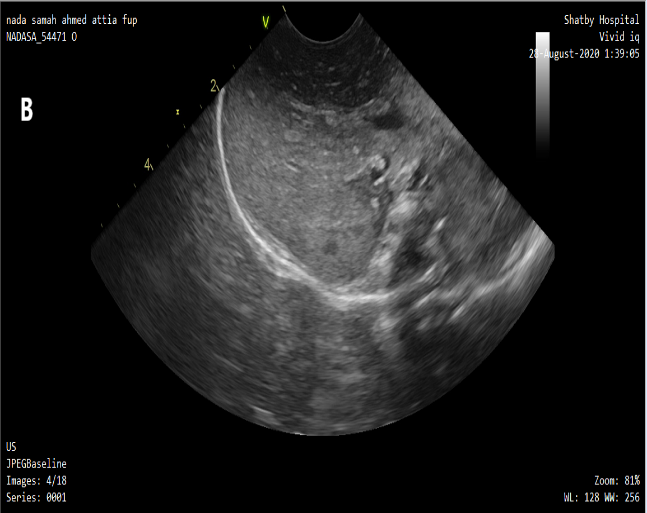


**Patient no.9 imaging:** Shows US images of a neonate with GA of 32 weeks with inserted femoral catheter. **A.** Lateral view shows an iso to hyperechoic thrombus (black arrows) around the catheter (white arrow points to the catheter tip). **B.** Axial view shows the thrombus in intrahepatic portion of IVC with partial occlusion of its lumen.


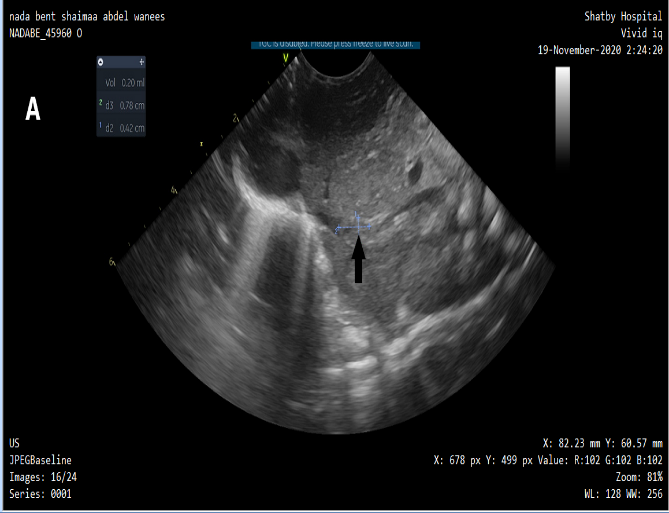

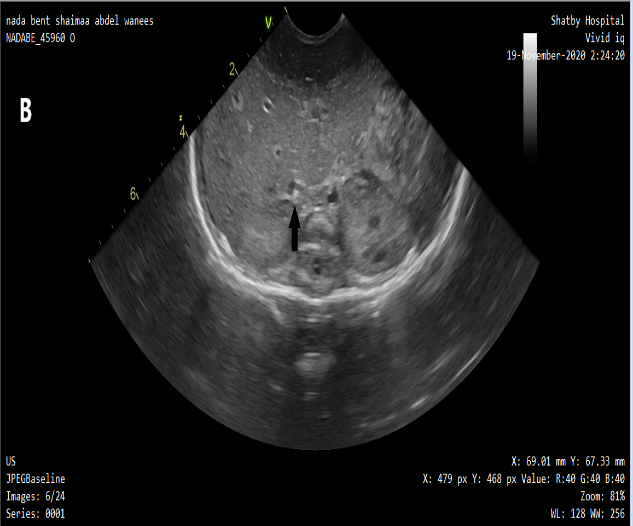


**Patient no.10 imaging:** Shows US images of a neonate with GA of 30 weeks with inserted femoral catheter. **A.** Sagittal view shows an isoechoic thrombus (black arrows) beyond the catheter tip. **B.** Axial view shows the thrombus in the intrahepatic portion of IVC with total occlusion of its lumen.


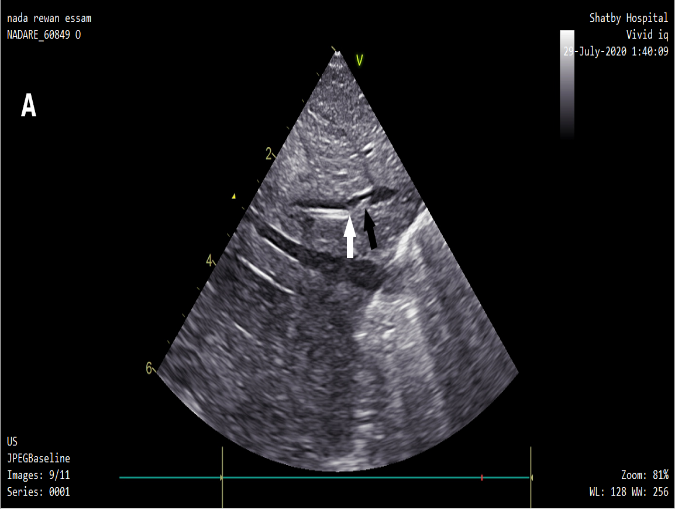

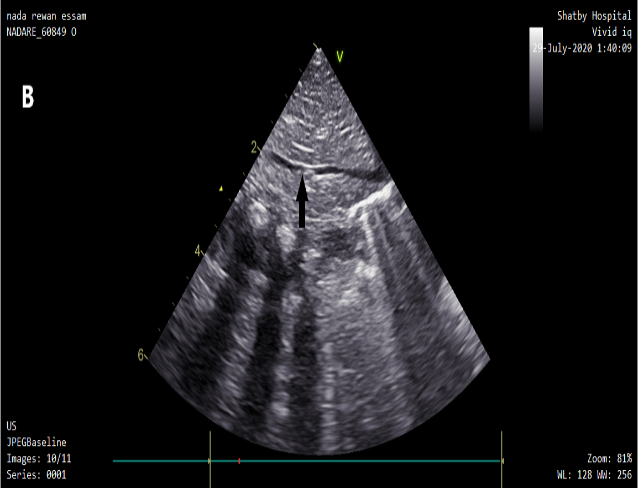


**Patient no.11 imaging:** Shows US images of a neonate with GA of 28 weeks with inserted femoral catheter. A small isoechoic thrombus (black arrows) is shown in IVC at the catheter tip (white arrow) with partial occlusion of its lumen.

**
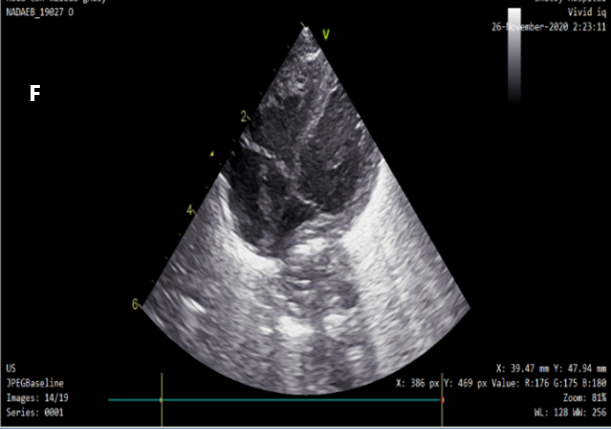

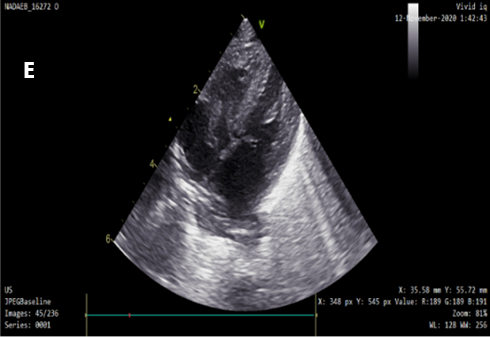

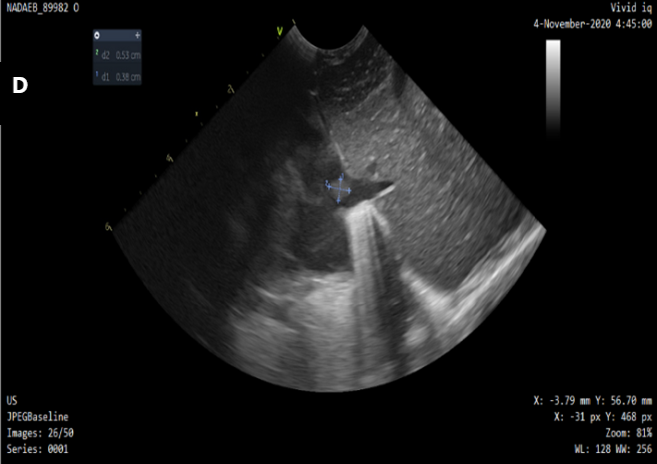
**
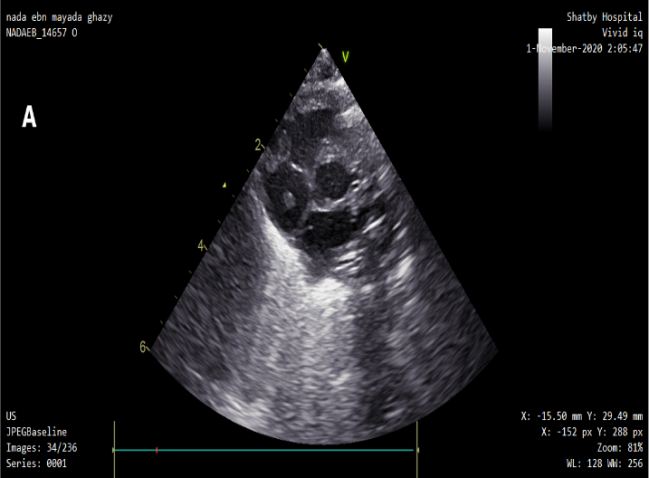

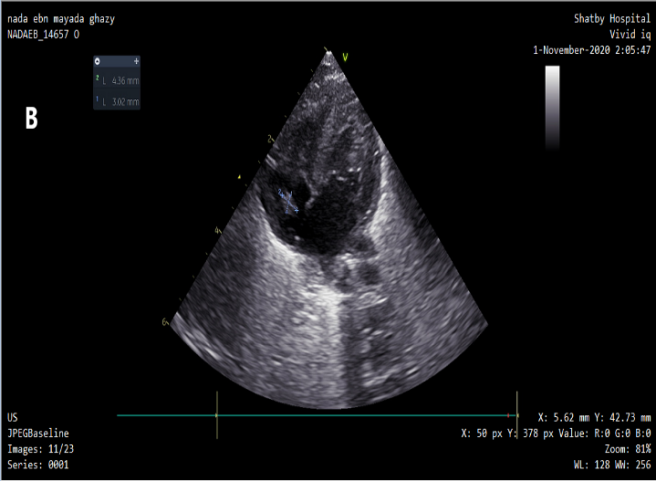


**
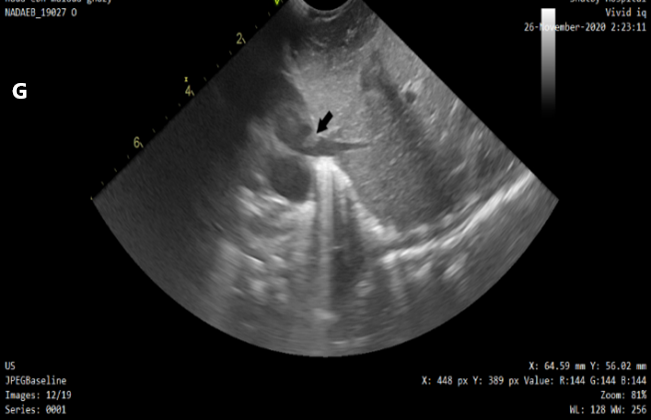

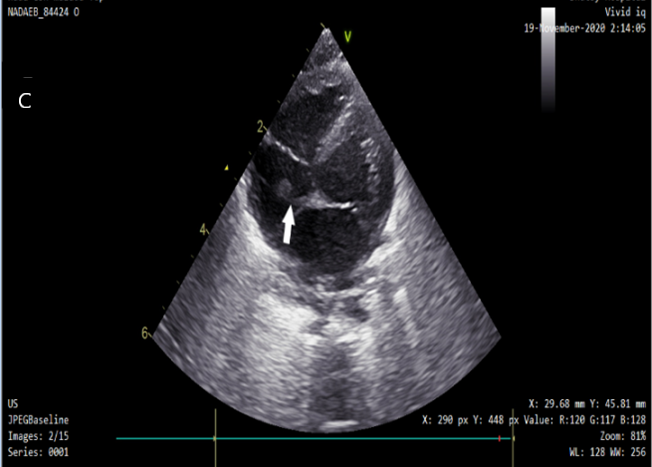
**

**Patient no.12 imaging:** Shows different US images of a neonate with GA of 34 week with thrombosis diagnosed after removal of UVC. **A.** Short axis parasternal view shows small thrombus in right atrium. **B.** Apical view showing size of the thrombus 4.3 mm x 3 mm. **C**. showing the mobile thrombus in right atrium with pedicle (white arrow) attached to interatrial septum. **D.** Another thrombus is noted on the same examination at right atrium/ IVC junction measuring 5.3 mm x 3.8 mm. **E.** On follow up examination, increase in size of the thrombus is noted and the thrombus extended through the tricuspid valve. **F**. the thrombus became beaded on follow up examination. **G.** shows decrease in size of Rt atrium/IVC junction thrombus (black arrow) but it is still present.

**
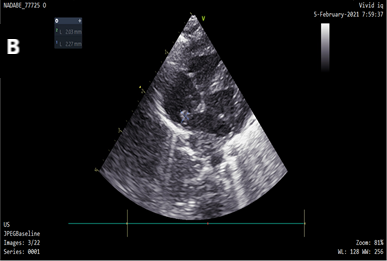

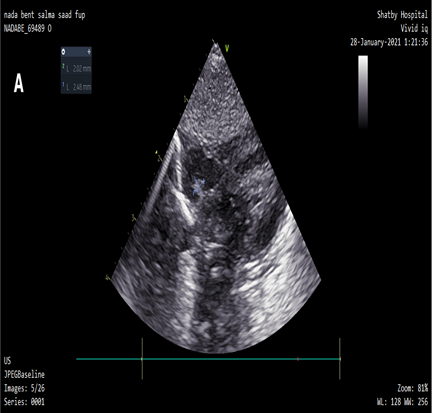
**

**Patient no.13 imaging:** Shows US images of a neonate with GA of 29 week with inserted malposed UVC (intracardiac traversing interatrial septum through PFO) **A.** Subcostal view shows small isoechoic intracardiac thrombus (black arrow) in right atrium measuring 2.4 mm x 2 mm. **B.** Apical view shows that the thrombus is still present on follow up examination.


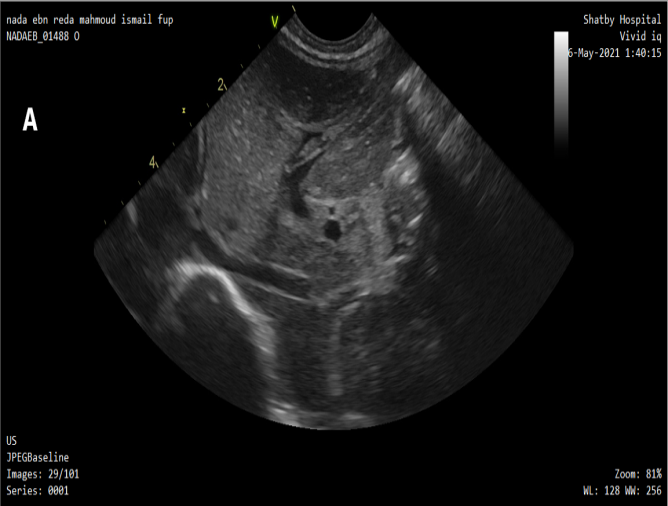

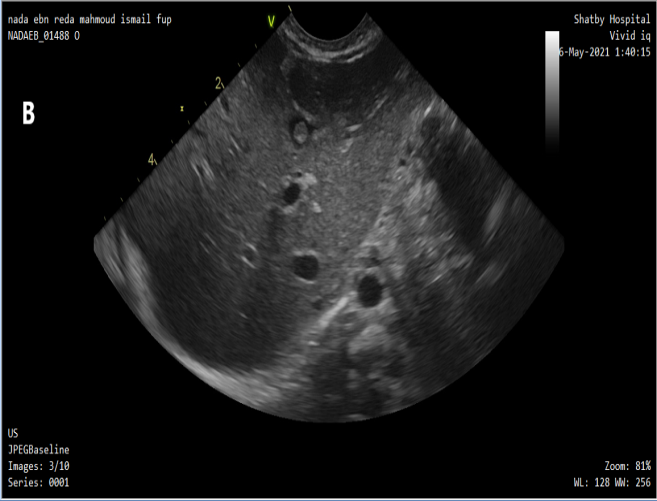


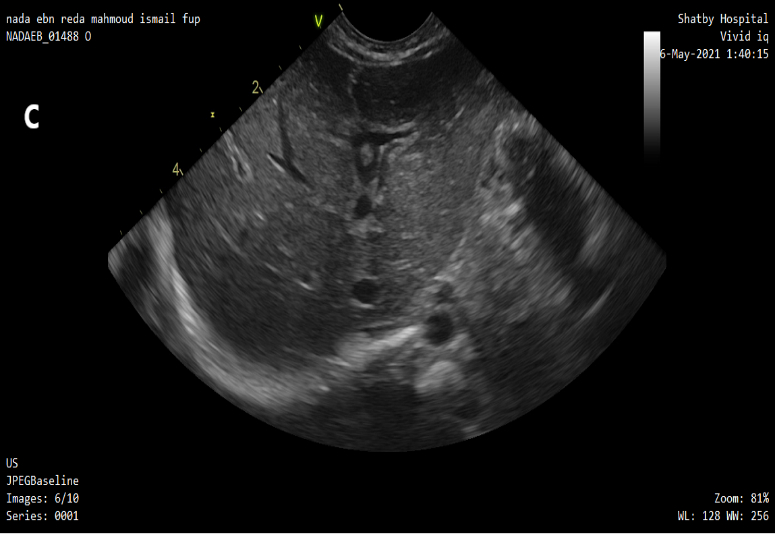


**Patient no.14 imaging:** Shows US images of a neonate with GA of 40 weeks and with inserted UVC after 4 days of catheter insertion. **A.** Sagittal view showing thrombus around the catheter. **B. &** **C.** Axial views showing the thrombus in umbilicoportal confluence.

**
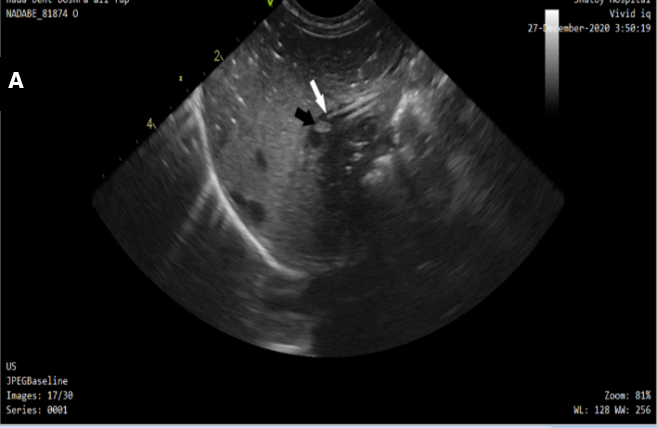
**

**
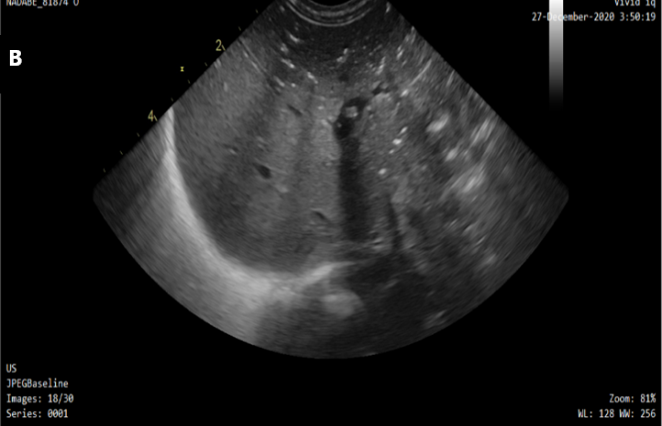
**

**
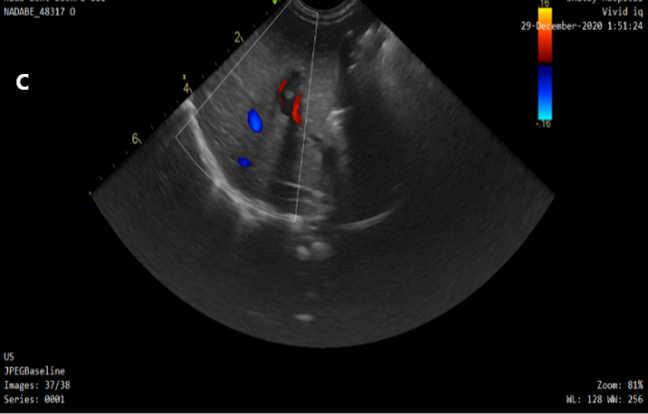
**

**Patient no.15 imaging:** Shows US images of a neonate with GA of 37 weeks and with inserted UVC after 9 days of catheter insertion. **A.** show small thrombus (black arrow) at the catheter tip (white arrow). **B.** Axial view shows presence of hyperechoic thrombus in umbilicoportal confluence. **C.** Color Doppler image shows presence of iso to hyper echoic thrombus at branching point of portal vein partially occluding its lumen with absence of flow on color doppler.


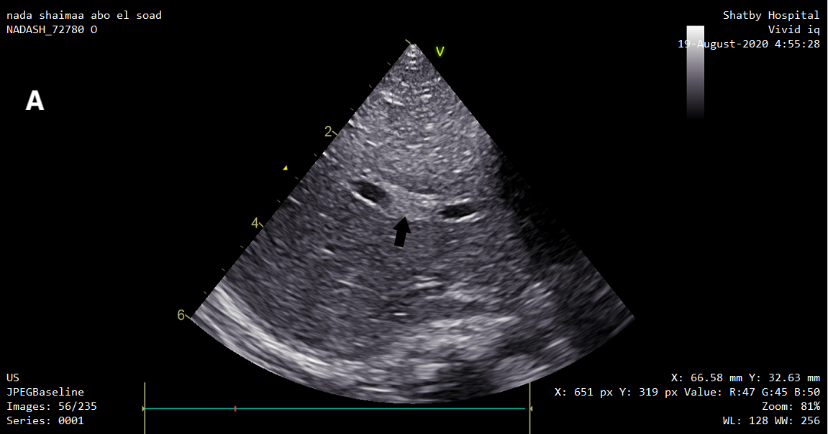


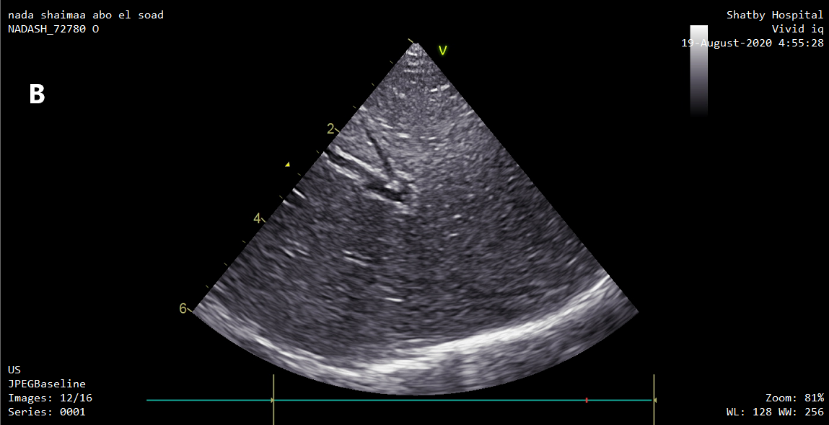


**Patient no.16 imaging:** Shows US images of a neonate with GA of 38 weeks and with inserted UVC after 3 days of catheter insertion. A thrombus is shown in umbilicoportal confluence in sagittal (**A**) and axial (**B**) views.


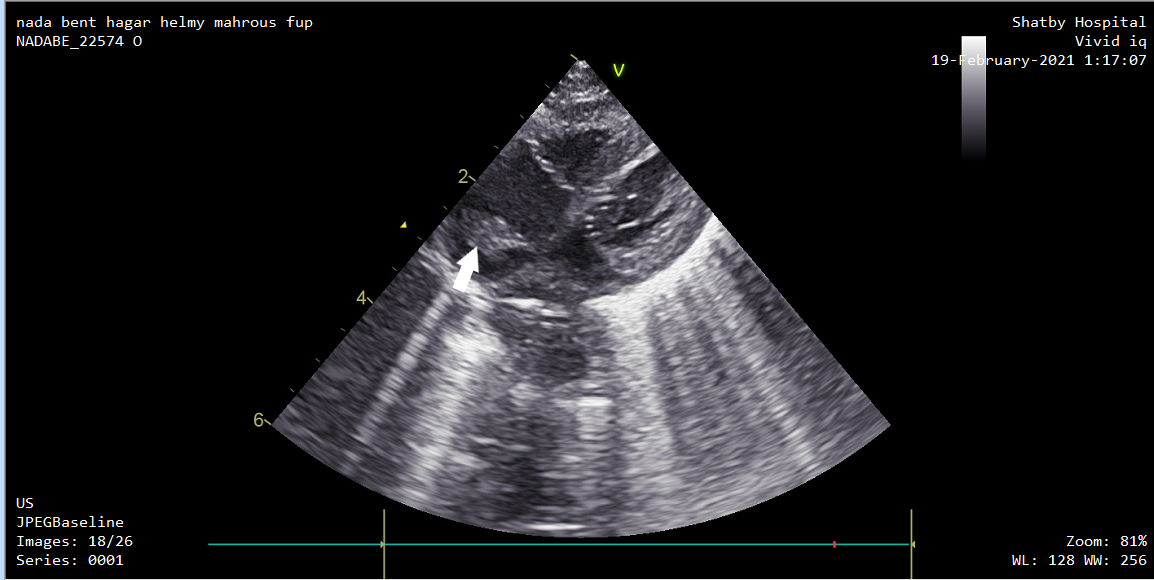


**Patient no.17 imaging:** Shows US image of a neonate with GA of 31 weeks and with inserted internal jugular central catheter after 9 days of catheter insertion. A large isoechoic thrombus (white arrow) is seen in the right atrium.
